# The E3 Ubiquitin Ligase ARIH1 Facilitates Colorectal Cancer Progression by Promoting Oxidative Phosphorylation via the Mitochondrial Translocation of K63‐Linked Ubiquitinated PHB1

**DOI:** 10.1002/advs.202501017

**Published:** 2025-04-26

**Authors:** Ying Tong, Zhenling Wang, Yong Wang, Yang Chen, Hongqiang Zhang, Yunfei Lu, Lei Xu, Hengyang Shen, Changzhi Huang, Min Zhao, Wenjie Li, Shuai Wang, Yu Shao, Zan Fu

**Affiliations:** ^1^ Department of General Surgery The First Affiliated Hospital of Nanjing Medical University Nanjing Jiangsu 210029 China; ^2^ The Changzhou Maternal and Child Health Care Hospital Changzhou Medical Center Nanjing Medical University Changzhou Jiangsu 213000 China

**Keywords:** ARIH1, colorectal cancer, K63‐linked ubiquitination, mitochondrial translocation, oxidative phosphorylation, PHB1

## Abstract

The RBR E3 ubiquitin ligase ARIH1 has been proven to induce specific ubiquitylation of substrates, thereby regulating cell proliferation and the cell cycle. However, the understanding of how ARIH1 influence cancer development is limited. This study revealed that ARIH1 is upregulated in colorectal cancer (CRC) cells and facilitates cell growth and metastasis. Clinically, high ARIH1 levels are linked to an unfavorable CRC prognosis. Mechanistically, ARIH1 directly interacts with PHB1 via its RING1+RBR+RING2 domains, catalyzing the K63‐linked ubiquitination of PHB1 at lysine 186 (K186). The increased interaction between PHB1 and Akt through this modification results in PHB1 phosphorylation by Akt and its subsequent translocation into mitochondria, where it maintains mitochondrial stability and promotes oxidative phosphorylation (OXPHOS). Collectively, these findings demonstrate the role of ARIH1‐mediated K63‐linked ubiquitination of PHB1 in mitochondrial dynamics and OXPHOS, suggesting that it has potential as diagnostic biomarker and treatment target for CRC.

## Graphical abstract

The RBR E3 ubiquitin ligase ARIH1, which is upregulated in colorectal cancer cells, promotes cell growth and metastasis and correlates with an unfavorable CRC prognosis. Mechanistically, ARIH1 catalyzes K63‐linked ubiquitination of PHB1, enhancing the interaction between PHB1 and Akt. This leads to Akt‐mediated phosphorylation of PHB1 and its subsequent translocation into mitochondria, where it maintains mitochondrial stability and enhances oxidative phosphorylation.

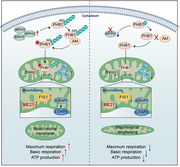

## 1 Introduction

Colorectal cancer (CRC) accounted for ≈1.9 million newly diagnosed cancer cases and 90000 related fatalities in 2022 and remains one of the most prevalent malignancies globally.^[^ ^1^ ^]^ The prognosis for advanced‐stage CRC patients remains poor, despite the emergence of novel therapies such as targeted therapies and immunotherapies.^[^ ^2^ ^]^ Recurrence and metastasis are the primary reasons for mortality in CRC patients, leading to a 5‐year survival rate of under 20% for these patients.^[^ ^3^ ^]^ Consequently, it is crucial to investigate the fundamental mechanisms of CRC progression and metastasis and to explore prospective biomarkers to guide clinical diagnosis and improve treatment.

Posttranslational modification (PTM), as an essential step in protein synthesis, affects the functions of proteins in various ways. PTMs, including ubiquitination, phosphorylation, SUMOylation, and acetylation, play crucial roles in various biological activities, such as cell growth, invasion, and death.^[^ ^4^ ^]^ Ubiquitination, as one of the most prevalent PTMs, can change the properties of some important substrate proteins, including activity, cellular localization, and interactions with other proteins.^[^ ^5^ ^]^ The process of ubiquitination involves three enzymes: an E1 ubiquitin‐activating enzyme, an E2 ubiquitin‐conjugating enzyme, and an E3 ubiquitin ligase.^[^ ^6^ ^]^ The different sites where ubiquitin molecules are linked to substrates determine the type of ubiquitination (including K11‐linked, K48‐linked, and K63‐linked ubiquitin) and result in different changes in the properties of the substrates.^[^ ^7^ ^]^ K48‐linked ubiquitination, the most common type of ubiquitin ligation, constitutes a critical component of the ubiquitin proteasome system (UPS), which mediates the proteolysis of substrate proteins.^[^ ^8^ ^]^ K63‐linked ubiquitination can influence protein‐protein interactions, activation, and translocation, processes that regulate tumor activity.^[^ ^9^ ^]^ In addition, ubiquitination can cooperate with other PTMs to affect downstream target proteins.

E3 ubiquitin ligases can be classified into three main types (HECT, RING, and RBR ligases) according to their special structural domain and the mechanism by which ubiquitin is transferred to substrate proteins.^[^ ^10^ ^]^ ARIH1, a vital member of the RBR subfamily that is highly expressed in several types of cancer cells, contains a RING1 domain, a RING2 domain and an in‐between‐RING (IBR) domain.^[^ ^11^, ^12^ ^]^ It has been reported that ARIH1 can modulate tumor progression through activating epithelial‐to‐mesenchymal transition (EMT) by regulating hnRNP E1 protein stability in breast cancer.^[^ ^13^ ^]^ In addition, ARIH1 can mediate mitophagy, which contributes to therapeutic resistance in cancer cells.^[^ ^14^ ^]^ Furthermore, ARIH1 accumulates in perinuclear areas and induces 4EHP‐mediated translational arrest after DNA damage to protect cancer cells from genotoxic stress.^[^ ^15^ ^]^ Nonetheless, studies on the role of ARIH1 in CRC are limited.

The number and morphology of mitochondria, highly dynamic organelles found in most eukaryotic cells, constantly change throughout the processes of fusion and fission. These mitochondrial dynamics help maintain optimal oxidative phosphorylation (OXPHOS) activity.^[^ ^16^, ^17^ ^]^ Several decades ago, Warburg discovered that the level of glycolysis in tumor cells was higher than that in normal cells, resulting in mitochondrial damage and OXPHOS suppression in cancer cells.^[^ ^18^ ^]^ However, recent experimental evidence indicates that mitochondrial respiratory function and OXPHOS activity are also increased in some cancers, exerting a significant impact on cell proliferation, tumor metastasis, and drug resistance.^[^ ^19^ ^]^ The level of OXPHOS is heterogeneous among different tumors and even within the same tumor.^[^ ^20^ ^]^ Consequently, additional studies are required to explore the impact of OXPHOS and mitochondrial function on CRC progression.

Prohibitin 1 (PHB1), a member of the prohibitin family, is widely distributed across various cellular compartments, including mitochondria, the nucleus, and the cell membrane, and its localization depends on its PTMs.^[^ ^21^ ^]^ Notably, PHB1 has diverse functions that depend on its subcellular localization. In mitochondria, PHB1 is located in the inner membrane and regulates mitochondrial fusion and fission by stabilizing optic atrophy protein 1 (OPA1) and dynamin‐related protein 1 (DRP1).^[^ ^22^ ^]^ Moreover, PHB1 regulates OXPHOS activity and promotes mitochondrial respiration by modulating the stability of OXPHOS complexes.^[^ ^23^, ^24^ ^]^ Nevertheless, the molecular regulation of PHB1 in CRC is not yet fully understood.

Our study revealed that ARIH1 expression is high in CRC, which facilitates the growth and spread of CRC cells and is linked to an unfavorable prognosis. Mechanistically, ARIH1 facilitates the K63‐linked polyubiquitination of PHB1 by targeting the K186 residue, thereby promoting its interaction with Akt. This enables PHB1 to be phosphorylated by Akt and translocated into the mitochondria, where it stabilizes mitochondrial dynamics and increases mitochondrial respiration and OXPHOS activity.

## 2 Results

### 2.1 ARIH1 Exhibits High Expression Levels in CRC Tissues and Cell Lines, which is Associated with an Unfavorable Prognosis

**Figure 1:**
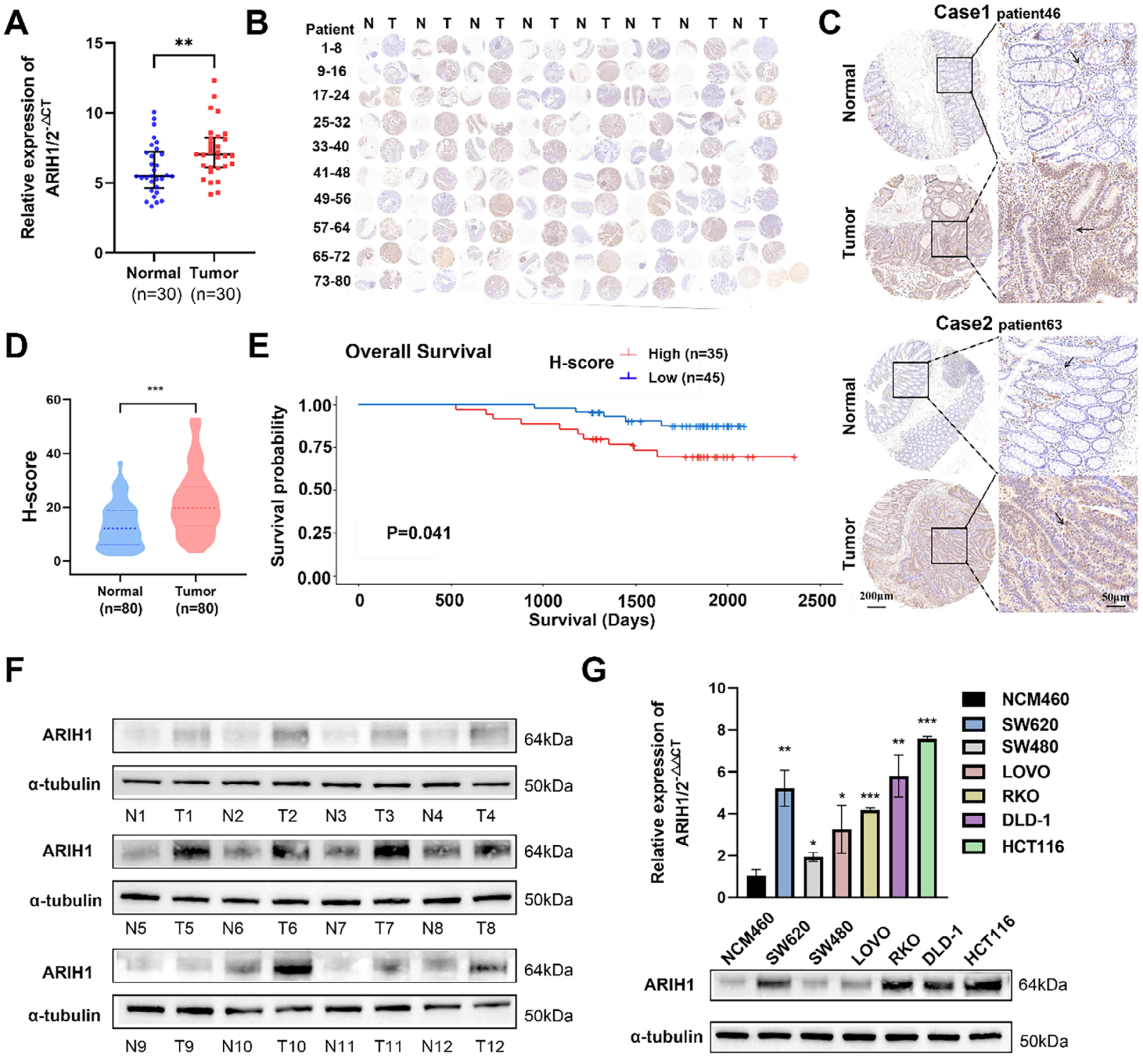
ARIH1 was highly‐expressed in CRC tissues and cell lines and correlated with poor prognosis. A) The ARIH1 mRNA expression levels were quantified by qRT‐PCR in 30 CRC tissues and matched adjacent normal tissues. B‐E) The protein level of ARIH1 detected by IHC in the TMA of 80 CRC patients’ samples. B) Comprehensive tissue microarray schema of ARIH1 expression, C) representative images of ARIH1 expression, D) the relative statistical analysis of H‐score, and E) the Kaplan‐Meier survival analysis of patients with high (n = 35) and low (n = 45) ARIH1 expression. F) The protein level of ARIH1 was detected by WB in 12 paired CRC tumor and adjacent tissues. G) The mRNA and protein levels of ARIH1 in CRC cell lines and NCM460 cells, evaluated by qRT‐PCR and WB respectively. Data was shown as mean±SD of three independent experiments, ^*^ *p *< 0.05, ^**^ *p *< 0.01, ^***^ *p *< 0.001.

To verify ARIH1 levels in CRC, we obtained 30 CRC tissue samples along with their corresponding normal tissues for measurement of the ARIH1 mRNA level via quantitative real‐time PCR (qRT‐PCR). We found that ARIH1 showed higher expression levels in tumor tissue (**Figure** **1**A), consistent with the results obtained from the TCGA database (Figure S1A, Supporting Information). Subsequently, tissue microarrays (TMAs) were constructed using samples from 80 patients (Figure 1B). Immunohistochemistry (IHC) analysis revealed high ARIH1 expression in CRC tissues (Figure 1C), with a significantly higher H‐score in tumor tissues (Figure 1D). Furthermore, the clinicopathological characteristics of these 80 patients were statistically analyzed and no correlations were detected between the ARIH1 level and age, sex, tumor site, tumor size, T stage, nerve invasion, or microsatellite stability. Nonetheless, significant associations were observed between the ARIH1 level and tumor stage (*p *< 0.001), distant metastasis (p = 0.012), vascular invasion (*p* = 0.013), and lymph node metastasis (*p *< 0.001) (**Table** **1**). These findings indicate that ARIH1 may be critically involved in the proliferation and metastasis of CRC. Moreover, samples from patients who had lymph node metastasis, distant metastasis, or vascular invasion had higher H‐scores (Figure S1B, Supporting Information). Overall survival (OS)analysis via the Kaplan‐Meier method revealed that high ARIH1 expression was correlated with poor OS (*p* = 0.041) (Figure 1E), similar to the TCGA database analysis results (Figure S1C, Supporting Information). Twelve tissue samples were chosen for western blotting (WB), which revealed that ARIH1 was overexpressed in CRC tissues. Similarly, both the mRNA and protein levels of ARIH1 were greater in six CRC cell lines (SW620, SW480, RKO, LOVO, DLD‐1, and HCT116) than in normal colorectal mucosa epithelial cells (NCM460) (Figure 1F,G). In conclusion, ARIH1 expression is elevated in CRC and is correlated with disease progression, contributing to a poor patient prognosis.

**Table 1:** Analysis of the correlation between ARIH1 expression and clinicopathological features in 80 CRC patients.

Variable	All cases	ARIH1		*P* value
		High	Low	
**All Cases, n**	80	40	40	
**Age(years), n(%)**				
<60	25(31.3)	16(40.0)	9(22.5)	0.147
≥60	55(68.7)	24(60.0)	31(77.5)	
**Gender, n(%)**				
Male	51(63.8)	25(62.5)	26(65.0)	0.816
Female	29(36.2)	15(37.5)	14(35.0)	
**Tumor site, n(%)**				
Colon	35(43.8)	20(50.0)	15(37.5)	0.368
Rectum	45(56.2)	20(50.0)	25(62.5)	
**Tumor size(cm), n(%)**				
<5	32(40.0)	14(35.0)	18(45.0)	0.494
≥5	48(60.0)	26(65.0)	22(55.0)	
**TNM staging System, n(%)**				
T1‐2	17(21.3)	5(12.5)	12(30.0)	0.099
T3‐4	63(78.7)	35(87.5)	28(70.0)	
**Tumor stage, n(%)**				
StageI‐II	37(46.3)	9(22.5)	28(70.0)	<0.001^*^
StageIII‐IV	43(53.7)	31(77.5)	12(30.0)	
**Lymph node metastasis, n(%)**				
No	38(47.5)	10(25.0)	28(70.0)	<0.001^*^
Yes	42(52.5)	30(75.0)	12(30.0)	
**Vascular invasion, n(%)**				
No	67(83.8)	29(72.5)	38(95.0)	0.013^*^
Yes	13(16.2)	11(27.5)	2(5.0)	
**Nerve invasion, n(%)**				
No	69(86.3)	32(80.0)	37(92.5)	0.193
Yes	11(13.7)	8(20.0)	3(7.5)	
**Distant Metastasis, n(%)**				
No	73(91.3)	33(82.5)	40(100.0)	0.012^*^
Yes	7(8.7)	7(17.5)	0(0.0)	
**Cumulative survival, n(%)**				
No	15(18.8)	10(25.0)	5(12.5)	0.252
Yes	65(81.2)	30(75.0)	35(87.5)	
**Microsatellite(misssing, 28), n(%)**				
MSS	42(52.5)	20(50.0)	22(55.0)	0.991
MSI‐H	8(10.0)	4(10.0)	4(10.0)	
MSI‐L	2(2.5)	1(2.5)	1(2.5)	

TNM, Tumor node metastasis; MSS, microsatellite stability; MSI‐H, Microsatellite Instability‐High; MSI‐L, Microsatellite Instability‐Low. ^*^ *P* < 0.05 was considered significant.

### 2.2 ARIH1 Promotes the Proliferation and Metastasis of CRC Cells In Vitro

DLD‐1 and HCT116 cells, which present relatively high expression, and SW480 cells, which present the lowest expression of ARIH1 among the six CRC cell lines, were chosen for analysis of the functions of ARIH1. The overexpression vector for ARIH1 (ARIH1‐oe) was transfected into SW480 cells and three shRNAs targeting ARIH1 were introduced into DLD‐1 and HCT116 cells via lentiviral transduction to construct stable cell lines. The transfection efficiency was assessed via qRT‐PCR and WB (Figure S1D,E, Supporting Information). ShARIH1‐1 and shARIH1‐2 presented relatively high ARIH1 knockdown efficiencies and were thus used for subsequent experiments.

**Figure 2:**
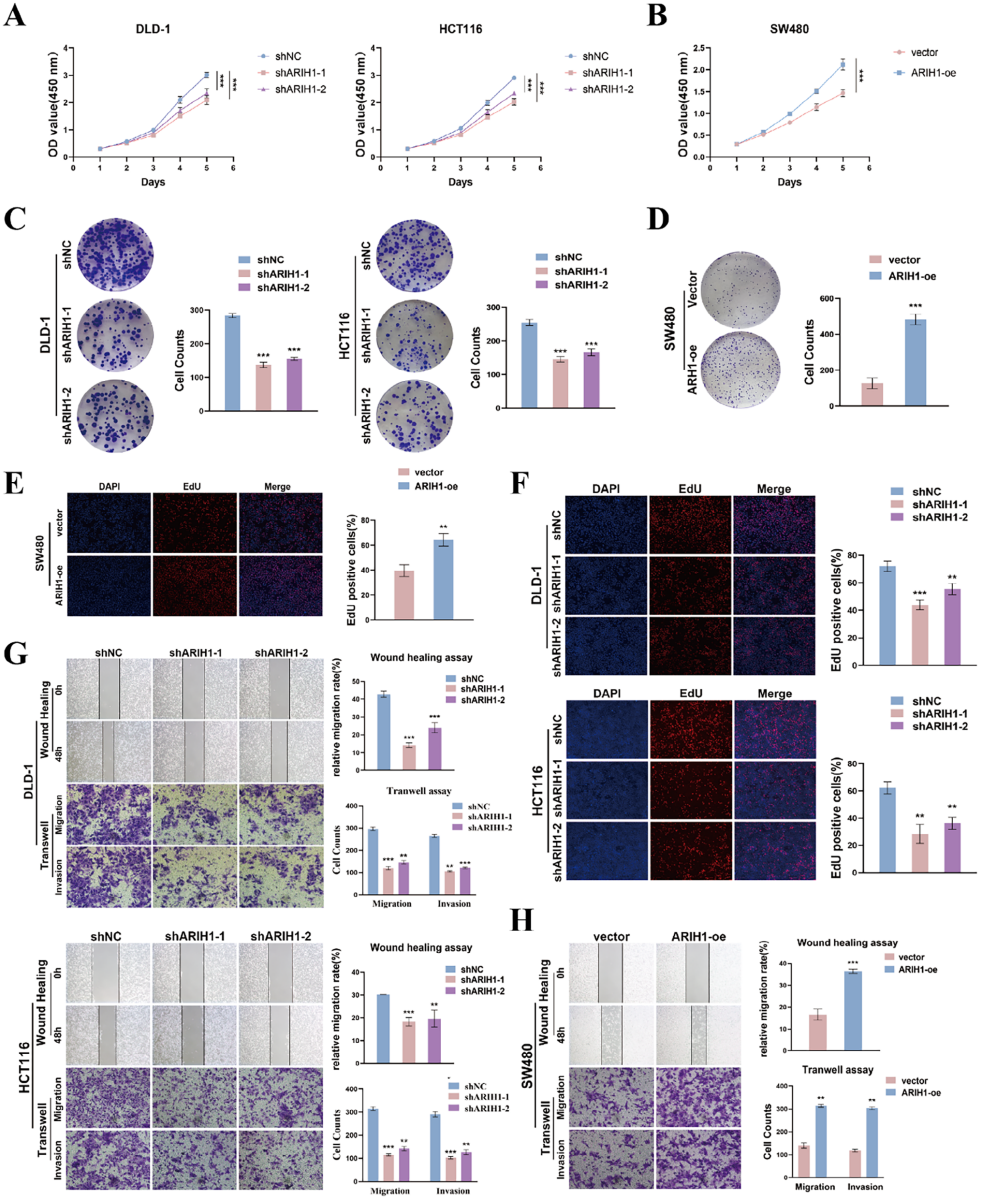
ARIH1 promotes the proliferation and metastasis of CRC cells in vitro. A,B) CCK8 assays were used to detect the viability of ARIH expression or knockdown cells. C,D) Colony formation assays were conducted to evaluate proliferation ability of SW480, DLD‐1, and HCT116 cells. E,F) EdU staining assays were performed to assess the cell proliferation ability. G,H) Transwell and wound healing assays were applied to evaluate migration and invasion abilities of CRC cells. Scale bar: 100µm. All data was shown as mean±SD of three independent experiments and was analyzed using either student's t‐test or ANOVA, ^*^ *p *< 0.05, ^**^ *p *< 0.01, ^***^ *p *< 0.001.

CCK‐8, colony formation, and EdU assays were performed to evaluate the ability of ARIH1 to affect cell proliferation. In SW480 cells overexpressing ARIH1, we observed accelerated growth, increased clonogenicity, and an increased percentage of EdU‐positive cells. Conversely, ARIH1 knockdown had the opposite effects on DLD‐1 and HCT116 cells (**Figure** **2**A–F).

Moreover, cell migration and invasion were assessed via Transwell and wound healing assays. The overexpression of ARIH1 in SW480 cells promoted the migration and invasion of CRC cells, whereas its downregulation suppressed these processes (Figure 2G,H). Taken together, these findings indicate that, in vitro, ARIH1 can promote CRC cell proliferation and metastasis.

### 2.3 ARIH1 Promotes the Proliferation and Metastasis of CRC Cells In Vivo

**Figure 3:**
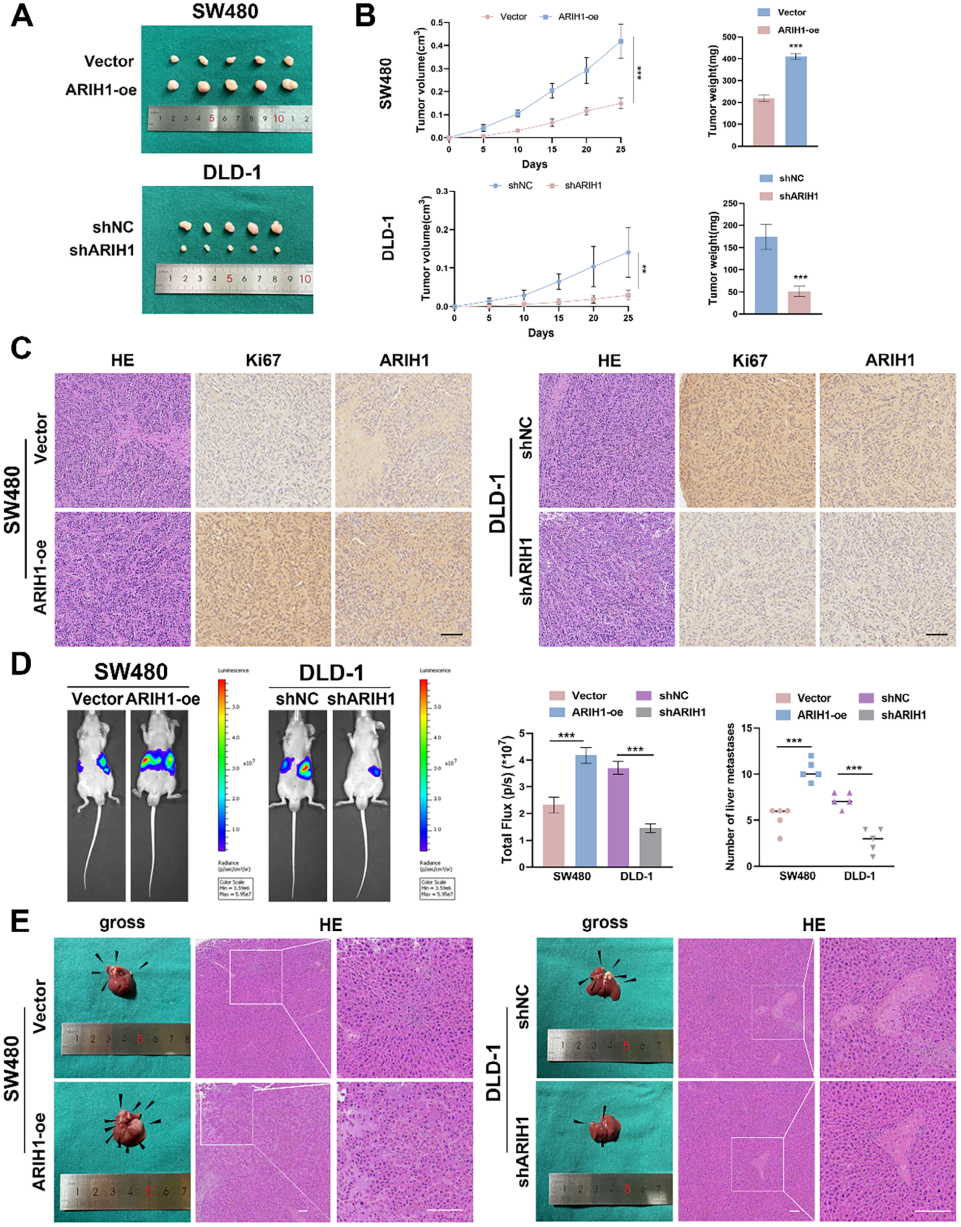
ARIH1 promotes the proliferation and metastasis of CRC cells in vivo. A–C) 1 × 10^6^ treated SW480 and DLD‐1 cells were injected subcutaneously into nude mice. A) Representative photographs pf subcutaneous xenograft tumors were obtained from nude mice, B) tumor volume (length × width^2^) and tumor weight were recorded and statistically analyzed. C) H&E and IHC staining of Ki67 and ARIH1 were performed in xenograft tumors. D,E) 1 × 10^6^ treated SW480 and DLD‐1 cells were injected into the spleen of nude mice. Black scale bar: 100µm. D) Representative images and analysis of bioluminescent intensity in liver metastases are shown. E) Representative photographs of liver metastases obtained from nude mice and H&E staining of liver metastatic tumors. The lesion indicated by the arrow represents a metastatic deposit in the liver. White scale bar: 100 µm. All data was shown as mean ± SD of three independent experiments and was analyzed using either student's t‐test or ANOVA, ^*^ *p *< 0.05, ^**^ *p *< 0.01, ^***^ *p *< 0.001.

Xenograft tumor models and liver metastasis models were developed to investigate the in vivo effects of ARIH1. In xenograft tumor models, SW480 cells, stably transfected with vector or ARIH1‐oe, or DLD‐1 and HCT116 cells transfected with shNC or shARIH1‐1 were injected into the nude mice subcutaneously. Tumor volume was assessed every 5 days, and tumor weight was determined 25 days post‐injection. The tumor volume and weight markedly increased when ARIH1 was overexpressed; however, inhibition of ARIH1 revealed an inverse effect (**Figure** **3**A,B). Furthermore, the protein level of Ki‐67, a marker of cell proliferation, and ARIH1 were evaluated via IHC staining, which showed relatively elevated Ki‐67 and ARIH1 expression in ARIH1‐oe group and the opposite result in shARIH1 group (Figure 3C).

For the liver metastasis model, pretreated SW480 and DLD‐1 cells were injected into the distal end of the spleen. We detected increased fluorescence intensity and an increased number of metastatic tumors in the ARIH1‐oe group, whereas the fluorescence intensity and the number of metastatic nodules in the liver decreased in the shARIH1 group (Figure 3D,E; Figure S1F, Supporting Information). Overall, in vivo, ARIH1 participates in the proliferation and metastasis of CRC cells.

### 2.4 ARIH1 Preserves Mitochondrial Dynamics and Increases Mitochondrial OXPHOS Activity

**Figure 4:**
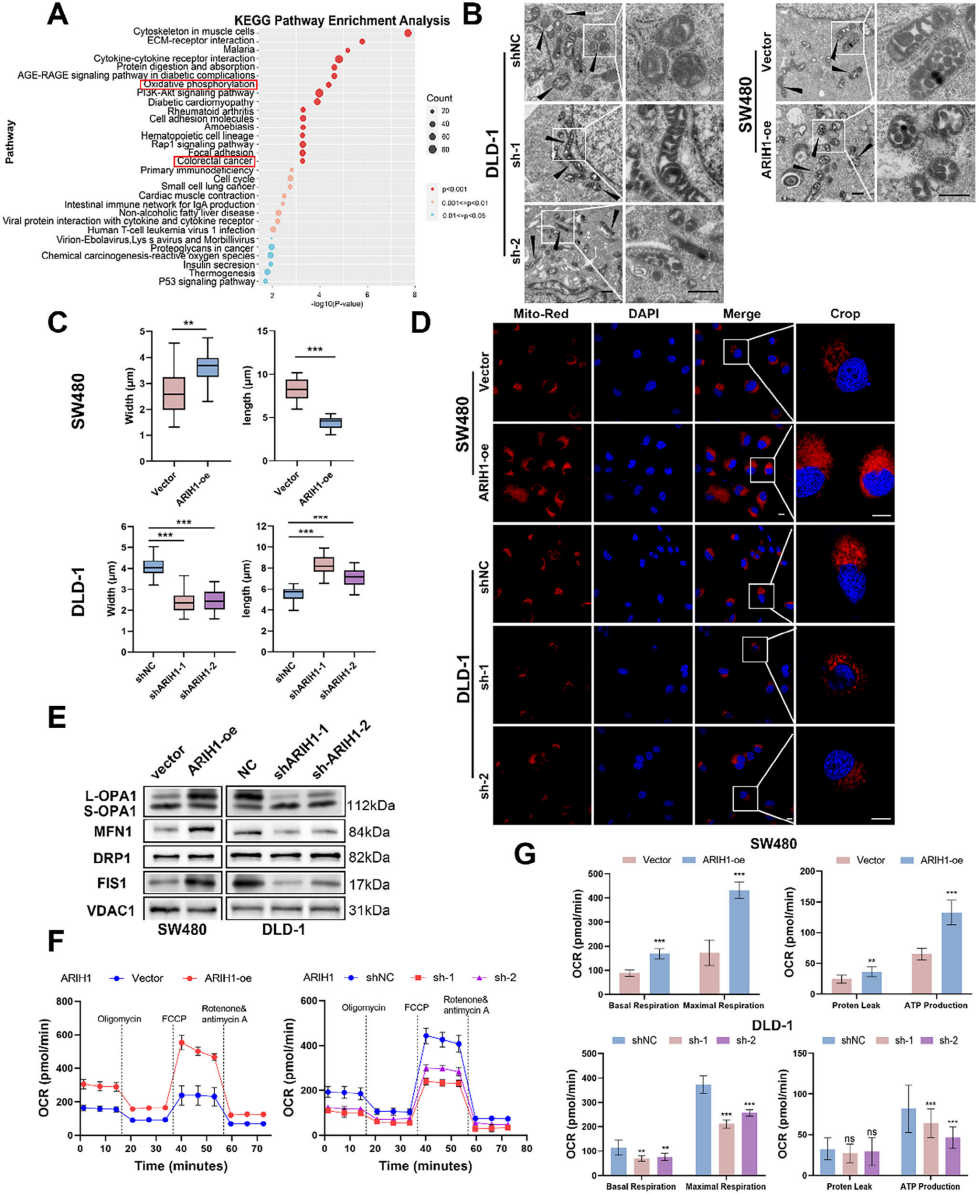
ARIH1 preserved mitochondrial dynamics and enhanced mitochondrial oxidative phosphorylation activity. A) KEGG analysis from the DEGs highly correlated with ARIH1 expression from bulk RNA‐seq. B) The morphology of mitochondria in ARIH expression or knockdown cells assessed by electron microscopy. Black scale bar: 0 .5µm. C) Width and length of mitochondria of SW480 and DLD‐1 cells were quantified and statistically analyzed. D) Representative images of Mitotracker Red assay in ARIH expression or knockdown cells to assess mitochondrial membrane potential. White scale bar: 10µm. E) Western blot analysis pf fusion (OPA1, MFN1) and fission (DRP1, FIS1) in CRC cells. F) The oxygen consumption rate (OCR) was measured with the Seahorse XF96 Analyzer in CRC cells applying mitochondrial stress test conditions. G) Basal respiration, maximal respiration, proton leak, and ATP production were measured and calculated. All data was shown as mean±SD of three independent experiments and was analyzed using either student's t‐test or ANOVA, ^*^ *p *< 0.05, ^**^ *p *< 0.01, ^***^ *p *< 0.001. FCCP: Carbonyl cyanide‐4‐(trifluoromethoxy) phenylhydrazone.

Bulk RNA sequencing was performed on ARIH1‐overexpressing cells and control cells, and the differentially expressed genes (DEGs) were identified to assess the mechanisms by which ARIH1 regulates CRC development. Most of the DEGs were enriched in oxidative phosphorylation pathways according to Kyoto Encyclopedia of Genes and Genomes (KEGG) pathway analysis (**Figure** **4**A), which is consistent with findings from the TCGA database (Figure S2A, Supporting Information). Therefore, we speculate that ARIH1 may influence CRC progression by modulating mitochondrial function.

To verify this hypothesis, we initially performed high‐resolution electron microscopy, which revealed that the mitochondria displayed longer and thinner structures in shARIH1 groups compared with the control groups, and shorter and wider structures in ARIH1‐oe cells than in control cells (Figure 4B,C; Figure S2B, Supporting Information). According to MitoTracker Red analysis, compared with control cells, ARIH1‐oe cells exhibited increased mitochondrial membrane potential, whereas shARIH1‐treated cells presented the decreased mitochondrial membrane potential, suggesting decreased mitochondrial stability in these cells (Figure 4D; Figure S2C, Supporting Information).

The expression of mitochondrial fusion and fission markers was analyzed by WB. In ARIH1‐overexpression cells, mitochondrial dynamin‐like GTPase OPA1 (OPA1) was transformed from a short isoform to a long isoform, and Mitofusin1 (MFN1) and Fission 1 (FIS1) levels are markedly increased. In shARIH1‐transfected cells, the reverse process was observed for OPA1 and there was a significant reduction in MFN1 and FIS1 expression, indicating that the molecular machineries of mitochondrial fusion and fission were impaired (Figure 4E; Figure S2D, Supporting Information).

Furthermore, the oxidative respiratory function of mitochondria was assessed. Through a mitochondrial stress test, we detected an increased OCR in the SW480 ARIH1‐oe cell line. In contrast, DLD‐1 and HCT116 cells with ARIH1 knockdown presented a decreased OCR (Figure 4F; Figure S2E, Supporting Information). Similarly, basal respiration, maximal respiration, and ATP production remarkably increased in SW480 cells but decreased in DLD‐1 and HCT116 cells (Figure 4G; Figure S2E, Supporting Information). After the normalization of the data, we reached the same conclusions (Figure S2F, Supporting Information). Thus, ARIH1 maintains mitochondrial stability by promoting mitochondrial fusion and fission and facilitating mitochondrial respiration and OXPHOS.

### 2.5 ARIH1 Directly Interacts with PHB1 and Increases the Mitochondrial Protein Levels of PHB1

**Figure 5:**
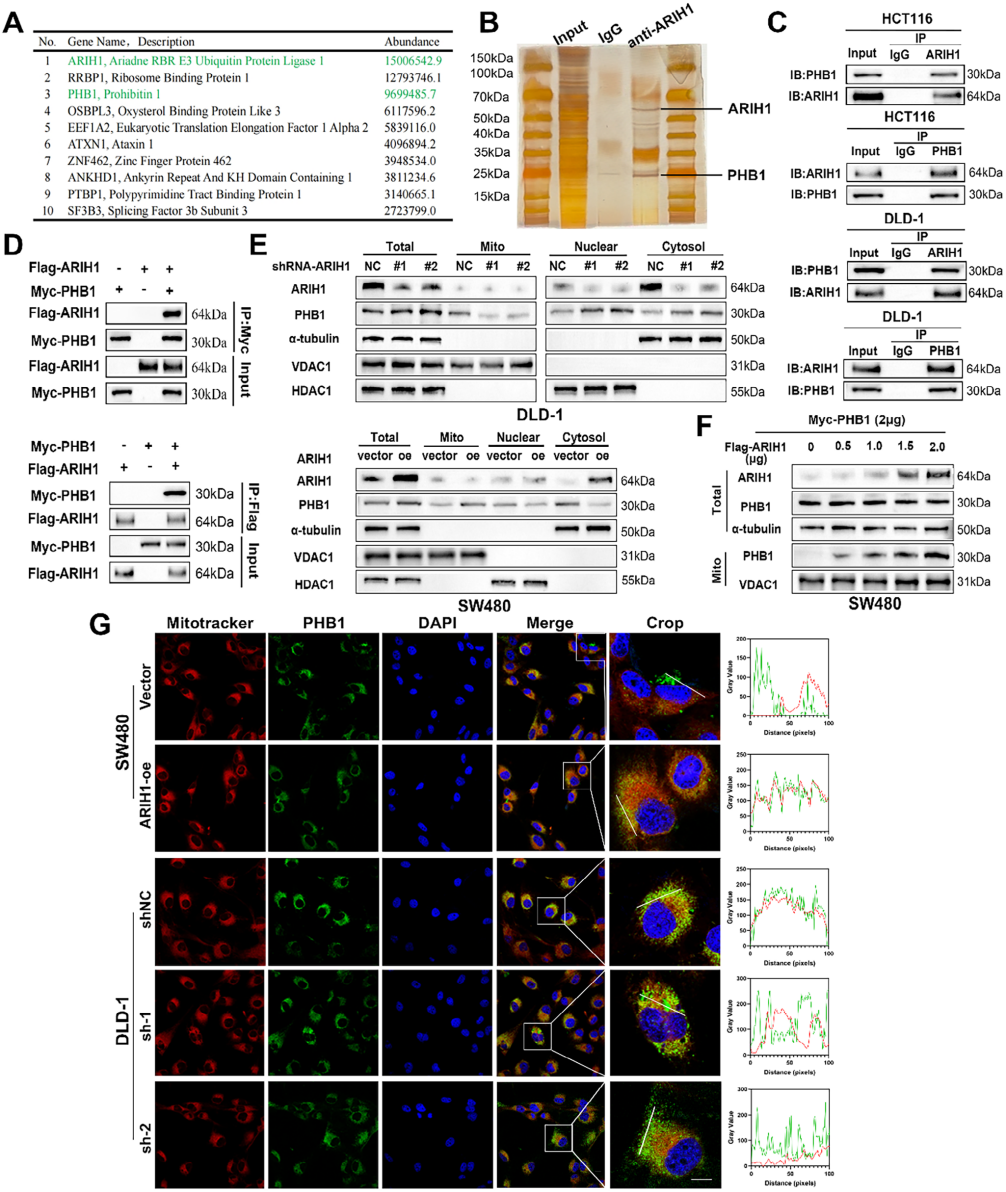
ARIH1 directly interacts with PHB1 and enhances the mitochondrial protein levels of PHB1. A) List of the top 10 proteins with the highest abundance value b mass spectrometry. B) Silver staining of ARIH1 immunoprecipitation lysates. The lines show different bands in immunoprecipitation assays between the ARIH1 group and IgG group. C) Lysates from DLD‐1 and HCT116 cells were analyzed by Co‐IP followed by Western blotting to investigate the endogenous interaction between ARIH1 and PHB1. D) Cell lysates from HEK293T cells transfected with the indicated plasmids for 24 h were analyzed by Co‐IP and Western blotting to investigate exogenous protein interactions. E) Protein levels of PHB1 in whole cells, nucleus, cytoplasm (excluding mitochondria), and mitochondria extracts were assessed by Western blotting in ARIH1‐overexpressing and ARIH1‐knockdown cells. α‐tubulin, VDAC1, and HDAC1 served as a loading control. F) SW480 cells were transfected with plasmids encoding Myc‐tagged PHB1 and varying amounts of Flag‐tagged ARIH1 for 24 h. Cell lysates were subsequently analyzed by Western blotting using the indicated antibodies. G) Representative images of PHB1 and MitoTracker Deep Red FM immunofluorescence staining in SW480 and DLD‐1 cells obtained to assess the co‐localization of PHB1 with the mitochondrial marker. Intensity profiles (right panel) were derived from the cropped images, indicated by a small white line. Red fluorescence corresponds to the mitochondrial marker, while green fluorescence denotes PHB1. Scale bar: 10µm. The results are representative of three independent experiments.

To elucidate the underlying molecular mechanism by which ARIH1 promotes CRC progression by affecting mitochondrial function, proteins that interact with ARIH1 were identified via immunoprecipitation (IP) analyses and mass spectrometry. The top ten proteins with the highest abundance values are listed; among these proteins, PHB1, which was previously reported to affect mitochondrial dynamics and OXPHOS attracted our attention (**Figure** **5**A). Silver staining revealed several specific bands of proteins in the ARIH1‐immunoprecipitated group compared to the IgG group; one band represented PHB1 at a molecular weight of ≈30 kDa (Figure 5B). To confirm the physical interaction between ARIH1 and PHB1, an endogenous co‐immunoprecipitation (Co‐IP) assay was conducted, which showed ARIH1 and PHB1 were pulled down by each other but not by IgG in DLD‐1 and HCT116 cells, indicating the endogenous interactions between ARIH1 and PHB1 (Figure 5C). Next, am exogenous Co‐IP assay was performed in HEK‐293T cells transfected with both Flag‐ARIH1 and Myc‐PHB1. High Myc‐PHB1 protein expression was detected with an anti‐Flag antibody, and high Flag‐ARIH1 expression was detected with an anti‐Myc antibody (Figure 5D). These results indicate that ARIH1 can interact with PHB1 both endogenously and exogenously.

As ARIH1 is an E3 ubiquitin ligase that participates in ubiquitination which can affect the interaction, translocation and activation of protein substrates, we assumed PHB1 as a ubiquitination substrate of ARIH1. Hence, ARIH1 was overexpressed or knocked down respectively to study whether ARIH1 can regulate the expression level of PHB1. The results showed that ARIH1 did not alter the mRNA expression level of PHB1, which confirmed that ARIH1 modulates PHB1 expression at the post‐transcription level (Figure S3A, Supporting Information). Next, we observed that neither the overexpression nor the knockdown of ARIH1 led to a change in endogenous PHB1 protein expression. Therefore, we further isolated the nuclear, mitochondrial and cytoplasmic (excluding mitochondrial) proteins from the above cells and detected the protein content of PHB1 by WB. Surprisingly, elevated PHB1 protein levels in mitochondria and a reduction of PHB1 protein levels in the cytoplasm and nucleus were observed in ARIH1‐expressing cells. The opposite results were also observed in the ARIH1‐knockdown cells (Figure 5E; Figure S3B, Supporting Information). Consistent with these findings, in SW480 cells, ectopic expression of ARIH1 increased the mitochondrial protein level of PHB1 in a dose‐dependent manner (Figure 5F). Additionally, an endogenous Co‐IP assay was conducted on the isolated cytoplasm of DLD‐1 and HCT116 cells, which showed that the interaction of ARIH1 and PHB1 occurred mainly in the cytoplasm (Figure S3C, Supporting Information). On the basis of these findings, we hypothesized that ARIH1 promotes the translocation of PHB1 into the mitochondria. To further validate this hypothesis, an immunofluorescence (IF) assay was performed to observe the local co‐localization of PHB1 and mitochondria. Consistent with our WB results, PHB1 protein levels remained unchanged in both the ARIH1‐overexpressing and ARIH1‐knockdown cells. Interestingly, in SW480 cells in which ARIH1 was overexpressed, the co‐localization of PHB1 with mitochondrial markers was enhanced, and the knockdown of ARIH1 weakened the co‐localization of PHB1 and mitochondrial markers (Figure 5G; Figure S3D, Supporting Information). Thus, ARIH1 can interact with PHB1 and promote the translocation of PHB1 into mitochondria.

### 2.6 ARIH1 promotes the K63‐linked polyubiquitination of PHB1 by targeting the K186 residue, a process that requires the RING1, RBR, and RING2 domains of ARIH1 as well as the coiled‐coil (CC) domain of PHB1

**Figure 6:**
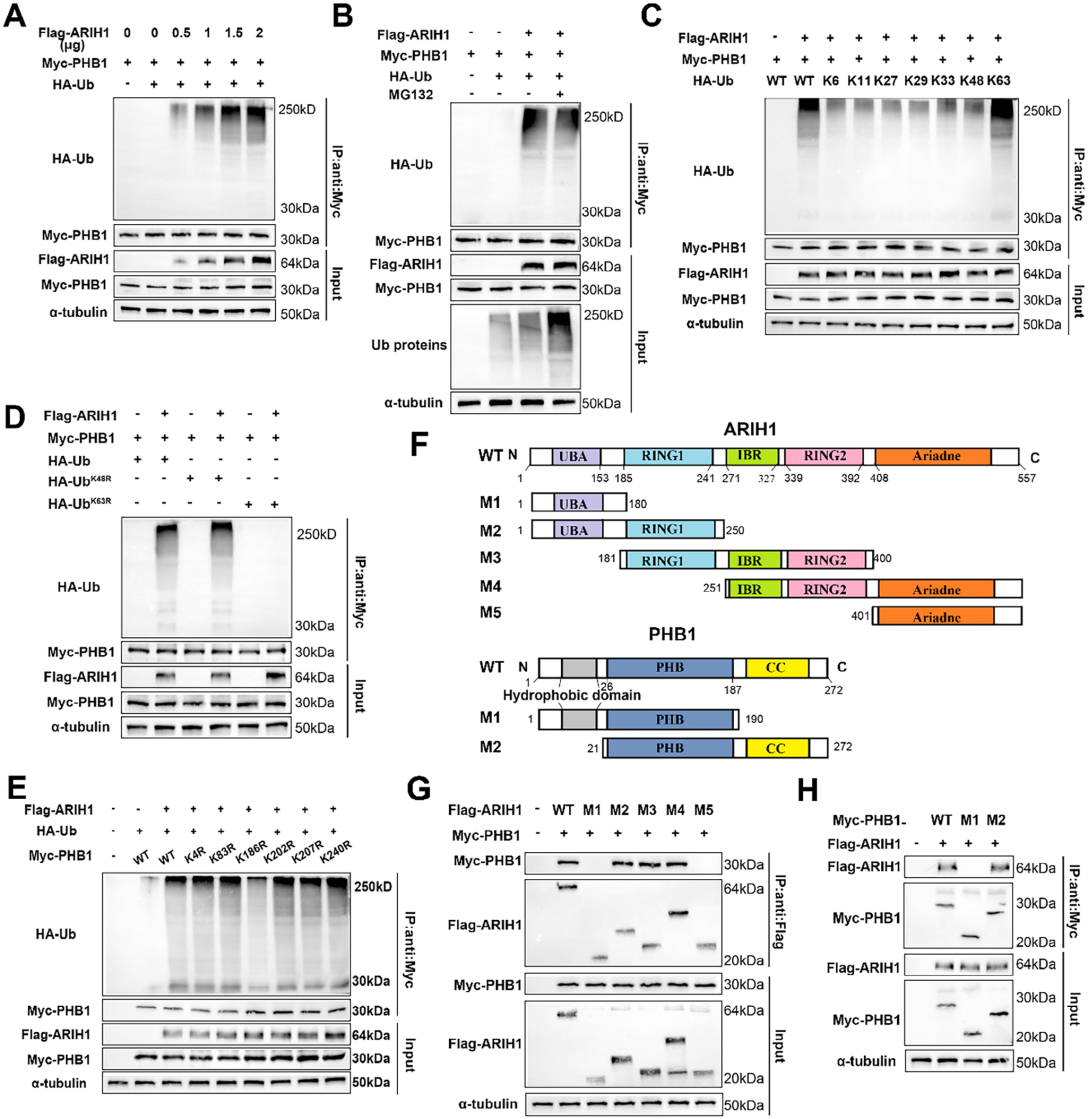
ARIH1 promotes the K63‐linked polyubiquitination of PHB1 by targeting the K186 residue, a process that requires the RING1, RBR, and RING2 domains of ARIH1 as well as the CC domain of PHB1. A) Plasmids encoding Myc‐tagged PHB1, HA‐tagged Ub, and increasing amounts of Flag‐tagged ARIH1 were transfected into HEK293T for 24 h. Cell lysates were analyzed by western blotting to detect ubiquitination levels of PHB1. B) HEK293T cells transfected with aforementioned plasmids were treated with MG132 (10µM) for 8 h, and the ubiquitination levels were analyzed by western blotting. C,D) HEK293T cells were transfected with Flag‐ARIH1, Myc‐PHB1, and wild‐type (WT) or mutant ubiquitin as indicated. Cell lysates were analyzed by western blotting. E) HEK293T cells transfected with Flag‐ARIH1, HA‐Ub, and WT or mutant PHB1 plasmids as indicated were subsequently harvested for western blot analysis. F) Schematic illustration of the full length ARIH1 and PHB1 and their deletion mutations. G,H) HEK293T cells were transfected with the specified plasmids. After 24 h, cell lysates were harvested and analyzed by western blot using the indicated antibodies. G) Plasmids encoding Myc‐PHB1, together with plasmids encoding either wild‐type Flag‐ARIH1 or its mutants (M1, M2, M3, M4, M5) were transfected into 293T cells. H) Plasmids encoding Flag‐ARIH1, together with plasmids encoding either wild‐type Myc‐PHB1 or its mutants (M1, M2) were transfected into 293T cells. The results are representative of three independent experiments.

We next sought to explore the effect of ARIH1‐mediated ubiquitination of PHB1. Different concentrations of the Flag‐ARIH1 plasmid together with the Myc‐PHB1 and HA‐Ub plasmids were transfected into HEK293T cells, and ubiquitin protein levels were detected using an anti‐Myc antibody. We observed a dose‐dependent increase in the expression of the ubiquitination level of PHB1 (**Figure** **6**A). Similarly, endogenous ubiquitination experiments also demonstrated that the ubiquitination level of PHB1 was increased when ARIH1 was overexpressed and decreased when ARIH1 was knocked down (Figure S4A, Supporting Information). Subsequently, MG132, a proteasome inhibitor, was applied to HEK293T cells; it increased the expression of total ubiquitinated proteins in the cells, but the expression level of ubiquitinated PHB1 was unchanged (Figure 6B).

It was reported that ubiquitin molecules contain seven lysine sites (K6, K11, K27, K29, K33, K48, and K63). Among the types of ubiquitination, K48‐linked ubiquitination which is the most common type of ubiquitin linkage, is usually associated with the degradation of substrate proteins via the UPS. While K63‐linked ubiquitination can affect protein‐protein interactions and the activation of substrates.^[^ ^7^ ^]^ Therefore, to assess the ARIH1‐mediated ubiquitin linkage type on PHB1, a series of plasmids (containing the wide‐type sequence or seven mutants retaining one lysine residue with arginine replacement of the remaining residues) were transfected into HEK293T cells with Flag‐ARIH1 and Myc‐PHB1. It was found that ARIH1 could ubiquitinate PHB1 in the presence of the wild‐type and K63 ubiquitin (Figure 6C). Furthermore, K48R and K63R which K48 or K63 lysine was substituted by arginine were constructed. The K63R mutation significantly disrupted the polyubiquitination of PHB1 by ARIH1, whereas the K48R mutation caused no change in the ubiquitination level of PHB1 (Figure 6D). Therefore, ARIH1 mediates the non‐degradative, K63‐linked ubiquitination of PHB1.

Next, we intended to identify the potential ubiquitination site of PHB1 which participates in ARIH1‐mediated PHB1 ubiquitination. Considering the high conservation of ubiquitin‐modified lysine residues across eukaryotic species and the predictions made by GPS‐SUMO (http://sumosp.biocuckoo.org/index.php) for multiple lysine residues, a series of arginine‐to‐lysine substitution mutants of PHB1 (K4R, K83R, K186R, K202R, K207R, and K240R) were generated (Figure S4B, Supporting Information). The K186R mutation dramatically impaired the ubiquitination of PHB1 in the presence of ARIH1 (Figure 6E). Hence, ARIH1 induces the polyubiquitination of PHB1 by targeting the K186 residue.

To identify the structural domain required for the ARIH1‐PHB1 interaction, a group of ARIH1 and PHB1 deletion mutants were generated (Figure 6F). The wide‐type, M2, M3, and M4 ARIH1 mutants were able to bind with PHB1, indicating that the RING1, RBR, and RING2 domains (amino acids 181–400) were necessary for binding with PHB1 (Figure 6G). These three domains were also essential for ARIH1 to ubiquitinate PHB1, as conspicuous ubiquitination bands could be identified only when the M2, M3, and M4 ARIH1 mutant plasmids were co‐transfected with Myc‐PHB1 and HA‐Ub plasmids into HEK293T cells (Figure S4C, Supporting Information). Besides, we observed that the deletion of the CC domain (amino acids 191–272) in PHB1 disrupted the interaction between these two proteins, which showed that PHB1 bound to ARIH1 through its CC domain (Figure 6H). Taken together, the RING1, RBR, and RING2 domains of ARIH1 and the CC domain of PHB1 are required for ARIH1‐PHB1 interaction and ubiquitination.

### 2.7 ARIH1 Enhances the Interaction Between PHB1 and Akt, Facilitating the Phosphorylation of PHB1 by Akt and its Subsequent Translocation into the Mitochondria

**Figure 7:**
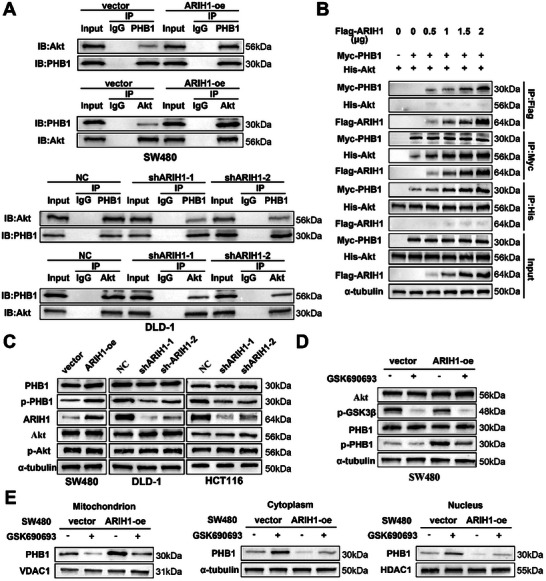
ARIH1 enhances the interaction between PHB1 and Akt, facilitating the phosphorylation of PHB1 by Akt and its subsequent translocation into the mitochondria. A) Lysates from ARIH1‐overexpression and ARIH1‐knockdown cells were analyzed by Co‐IP followed by Western blotting to investigate the endogenous interaction between PHB1 and Akt. B) Cell lysates from HEK293T cells transfected with the indicated plasmids for 24 h were analyzed by Co‐IP and Western blotting to investigate exogenous protein interactions. C) The protein levels of PHB1, p‐PHB1, Akt, and p‐Akt were evaluated by Western blotting in SW480, DLD‐1, and HCT116 cells stably transfected with the specified lentiviruses. D,E) SW480 cells were incubated with or without the Akt inhibitor GSK690693 (5 µm) for 12 h. Protein lysates from whole cells (D), nuclei, cytoplasm (excluding mitochondria), and mitochondria (E) were subsequently collected and subjected to Western blot analysis using the specified antibodies. The results are representative of three independent experiments.

As previously reported, Akt can bind with PHB1 and mediate its mitochondrial localization by phosphorylating it.^[^ ^25^ ^]^ Since ARIH1 interacted with PHB1 and promoted its translocation into mitochondria, we hypothesized that ARIH1 could increase Akt‐PHB1 interaction by promoting PHB1 ubiquitination. Endogenous immunoprecipitation of PHB1 and Akt was performed in ARIH1‐overexpressing SW480 cells and DLD‐1 and HCT116 cells with lentiviral transduction of shARIH1. ARIH1 overexpression increased PHB1/Akt complex formation, and ARIH1 deficiency decreased the connection between PHB1 and Akt (**Figure** **7**A; Figure S5A, Supporting Information). Furthermore, we transfected HEK293T cells with different doses of the Flag‐ARIH1 plasmid together with the Myc‐PHB1 and His‐Akt plasmids for an exogenous immunoprecipitation assay. These results indicated that the extent of the association between Akt and PHB1 is positively correlated with the concentration of ARIH1. Additionally, ARIH1 did not directly interact with Akt, but it facilitated the binding between Akt and PHB1 by modulating PHB1(Figure 7B). Consistently, PHB1, when mutated at the K186 ubiquitination site, lost its ability to bind to Akt (Figure S5B, Supporting Information), suggesting that the regulation of PHB1 ubiquitination by ARIH1 facilitates the interaction between PHB1 and Akt.

Previous studies have found that neither the overexpression nor the knockdown of ARIH1 leads to changes in endogenous PHB1 protein expression. Given that Akt can phosphorylate PHB1, we next assessed the expression levels of phosphorylated PHB1 in the aforementioned cells. As anticipated, the expression of phosphorylated PHB1 was also elevated in SW480 cells overexpressing ARIH1 and reduced in DLD‐1 and HCT116 cells with efficient ARIH1 knockdown (Figure 7C). Therefore, the Akt inhibitor GSK690693, a selective pan‐Akt kinase inhibitor which can suppress the phosphorylation of the Akt substrate GSK3β (Ser9) was used to inhibit the Akt‐mediated phosphorylation of PHB1. Treatment with GSK690693 significantly attenuated the ARIH1 overexpression‐induced increase in phosphorylated PHB1 expression in SW480 cells (Figure 7D). Nuclear, cytoplasmic (excluding mitochondrial), and mitochondrial proteins were isolated from SW480 cells overexpressing ARIH1 with or without GSK690693 treatment and analyzed via WB. The results indicated that the inhibition of Akt reduced the localization of PHB1 in the mitochondria and reversed the mitochondrial translocation of PHB1 induced by ARIH1 overexpression (Figure 7E).

Several phosphorylation sites of PHB1 have been reported.^[^ ^26^ ^]^ To identify the sites at which PHB1 is phosphorylated by Akt, a series of mutants in which threonine or serine residues were substituted with alanine residues (T258A, T108A, and S151A, Supporting Information) were generated (Figure S5C, Supporting Information). The T258A mutation but not the T108A or S151A mutation dramatically suppressed the phosphorylation of PHB1 (Figure S5D, Supporting Information). In summary, ARIH1 promotes the interaction between PHB1 and Akt, enabling PHB1 to be phosphorylated by Akt at the T258 site and subsequently translocated into the mitochondria.

## 3 Discussion

Accumulating evidence has demonstrated that ARIH1 interacts with components of the Cullin family, leading to the specific ubiquitylation of substrates and thereby regulating cell proliferation and the cell cycle.^[^ ^11^ ^]^ Additionally, ARIH1 participates in the activation of the host cell immune response.^[^ ^27^ ^]^ However, limited research has shown that ARIH1, an RBR E3 ubiquitin ligase, is involved in cancer development and progression. ARIH1 has been found to be highly expressed in various cancer cells, particularly in breast cancer and lung adenocarcinoma.^[^ ^14^ ^]^ ARIH1‐mediated mitophagy and ARIH1‐induced translational arrest, which are mediated by 4E‐homologous protein (4EHP), contribute to therapeutic resistance in cancer cells.^[^ ^14^, ^15^ ^]^ Our study reported that ARIH1 can promote the proliferation and metastasis of CRC cells by ubiquitinating the downstream protein PHB1, suggesting that ARIH1 may function as a promoting factor in cancer progression. Clinically, we observed that ARIH1 was overexpressed in CRC patients, and that increased ARIH1 expression was associated with unfavorable clinicopathological features and a poor prognosis. These results suggest that ARIH1 may function as a diagnostic and prognostic biomarker for CRC.

Mitochondria play crucial roles in various metabolic processes in tumor cells; therefore, alterations in mitochondrial function in cancer cells may contribute to cancer development.^[^ ^28^, ^29^ ^]^ Mitochondria are remarkably dynamic organelles, and the processes of fusion and fission, mediated by specific molecules such as OPA1, MFN1, DRP1, and FIS1, continuously regulate mitochondrial morphology, maintain mitochondrial DNA (mtDNA) integrity, and promote OXPHOS activity.^[^ ^30^, ^31^, ^32^ ^]^ Previous studies have reported that in lung cancer cells, the knockdown of the mitochondrial fission protein DRP1 leads to reduced proliferation and increased apoptosis.^[^ ^33^ ^]^ Moreover, OXPHOS plays a crucial role in cancer progression. In contrast to Warburg's earlier hypothesis that cancer cells exhibit increased glycolysis and decreased OXPHOS, recent studies have demonstrated that OXPHOS can be promoted to provide additional energy for cancer cell proliferation.^[^ ^34^ ^]^ The role of mitochondrial function in tumor research is indispensable. However, the impact of ubiquitination enzymes on tumor progression through their influence on mitochondrial function has been understudied. Parkinson protein 2 (PARK2), a well‐characterized RBR E3 ubiquitin ligase, has been found to exert tumor‐suppressive effects by inhibiting mitophagy, modulating glucose metabolism, and reducing mitochondrial respiration.^[^ ^35^, ^36^ ^]^ Elodie et al. identified that endogenous ARIH1 expression in lung cancer, breast cancer, and cervical cancer cell lines promotes the removal of damaged mitochondria via mitophagy, challenging the notion that the primary regulators of mitophagy are tumor suppressors.^[^ ^14^ ^]^ KEGG enrichment analysis of DEGs between bulk RNA‐seq data for ARIH1‐overexpressing cells and control cells revealed that ARIH1, which is also a member of the RBR E3 ubiquitin ligase family, is associated with OXPHOS. Therefore, we hypothesized that ARIH1 promotes CRC progression by regulating mitochondrial function. To investigate this hypothesis, we conducted a series of mitochondrial analyses, including electron microscopy for morphological analysis, MitoTracker Red staining, WB for fusion and fission‐related markers, and mitochondrial stress tests. Our findings indicate that ARIH1 is essential for maintaining mitochondrial stability and promoting mitochondrial respiration and OXPHOS. Compared to the findings of Elodie's study, both our results demonstrate that in various tumor cell types, ARIH1 regulates mitochondrial molecules, thereby influencing mitochondrial function. This observation underscores the potential significance of ARIH1's role in mitochondrial biology for cross‐tumor therapeutic strategies.

To further investigate the mechanism by which ARIH1 enhances CRC progression through its influence on mitochondrial function, we utilized Co‐IP and mass spectrometry to identify downstream proteins of ARIH1. PHB1 is essential for regulating cell cycle progression and is highly expressed in cervical, breast, and lung cancers.^[^ ^37^, ^38^, ^39^ ^]^ However, the role of PHB1 in cancer cell proliferation and tumor suppression is still a topic of debate. Studies have shown that PHB1 may function as a tumor suppressor through its co‐localization and interaction with the tumor suppressor proteins p53 and Rb, thereby increasing their transcriptional activity in the nucleus in breast and prostate cancers.^[^ ^40^ ^]^ Furthermore, Liu et al. reported reduced levels of PHB1 in gastric cancers. As a target of microRNA‐27a, which is upregulated in gastric cancer, PHB1 expression is diminished, thereby suppressing tumor development.^[^ ^41^ ^]^ Other studies have demonstrated that increased phosphorylation of PHB1 at T258 within the raft domain of the plasma membrane can activate the PI3K/Akt and Raf‐1/ERK signaling pathways, thereby promoting cancer progression.^[^ ^42^ ^]^ In cervical cancer cells, PHB1 functions as a chaperone or membrane anchor for C‐Raf, facilitating its interaction with Ras and thereby inducing cell migration through the activation of C‐Raf.^[^ ^43^ ^]^ Several anticancer drugs, such as rocaglamides and flavaglines, have been found to bind to PHB1, thereby preventing its interaction with C‐Raf and inhibiting C‐Raf activation.^[^ ^44^, ^45^ ^]^ PHB1, located in mitochondria, modulates the balance between mitochondrial fusion and fission events, maintains mtDNA integrity, and enhances membrane potential, ATP production, and oxygen consumption.^[^ ^46^, ^47^ ^]^ These conflicting results suggest that the subcellular localization of PHB1 may affect the role of PHB1 in tumorigenesis. Our study found that ARIH1‐mediated ubiquitination of PHB1 does not affect its expression levels but promotes its translocation into mitochondria to exert its functional role. These findings diverge from those of prior studies and may indicate unique mechanisms specific to CRC. Consequently, for effective tumor treatment, more in‐depth research and precise targeting strategies for PHB1 are essential, given its distinct functional roles in various tumor types, which are influenced by its subcellular localization.

Moreover, rather than the traditional K48‐linked ubiquitin proteasome degradation pathway, ARIH1 catalyzes the K63‐linked polyubiquitination of PHB1 at K186. K63‐linked ubiquitination can modulate protein properties such as protein‐protein interactions, translocation, and activation.^[^ ^48^ ^]^ Previous studies have reported that K63‐linked polyubiquitination of ERK by TRIM15 promotes its interaction with and activation by MEK.^[^ ^49^ ^]^ Previous studies have shown that Akt phosphorylates PHB1, inducing its mitochondrial localization and promoting bladder cancer cell proliferation.^[^ ^25^, ^50^ ^]^ The results of Co‐IP and pull‐down assays confirmed our hypothesis that K63‐linked ubiquitination of PHB1 mediated by ARIH1 enhances the association between PHB1 and Akt, which in turn triggers the phosphorylation of PHB1 at T258 and its translocation to mitochondria. Treatment with an Akt inhibitor decreased phosphorylated PHB1 levels and reversed the mitochondrial translocation of PHB1 induced by ARIH1 overexpression.

## 4 Conclusion

**Figure 8:**
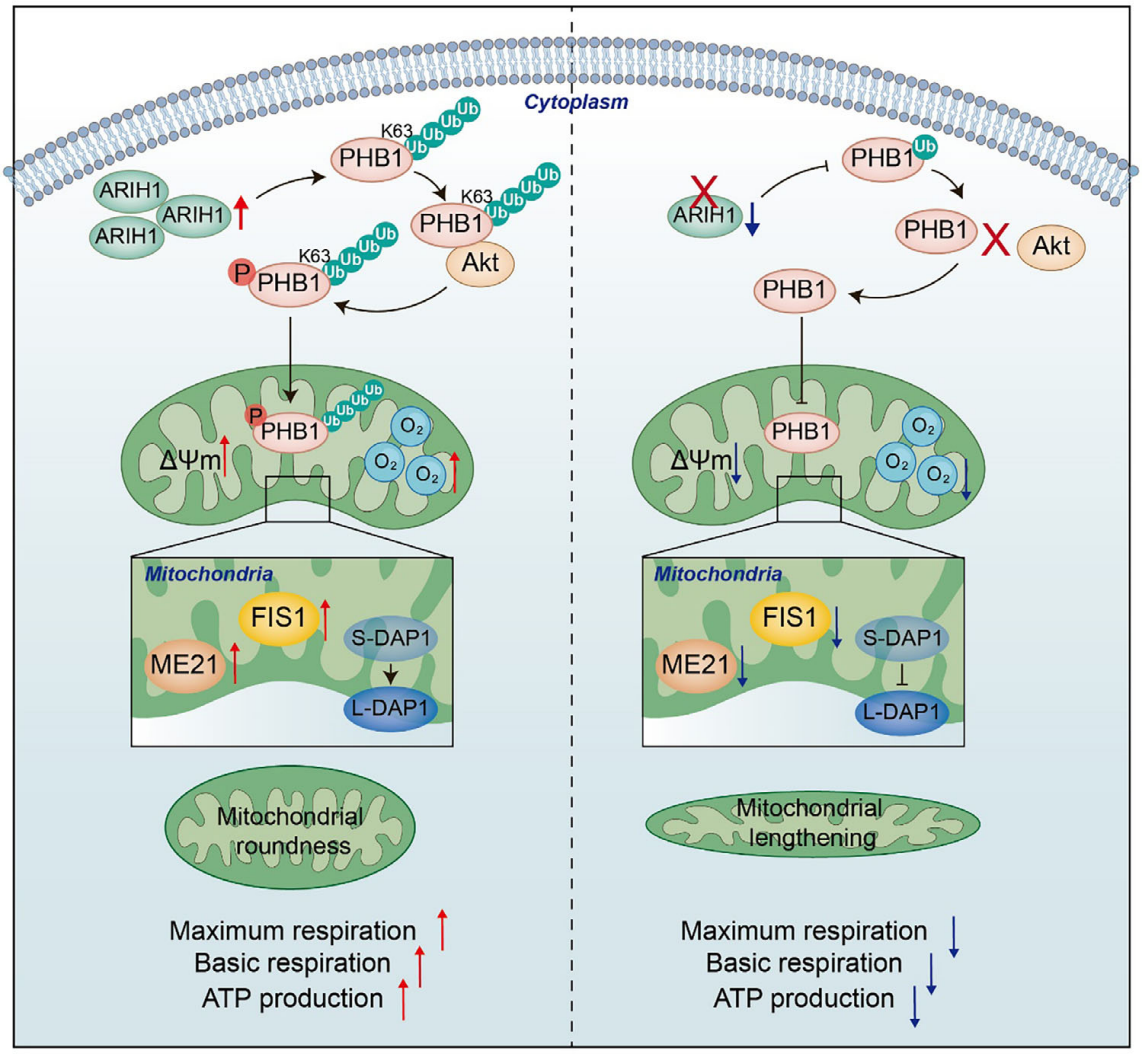
A schematic model for the mechanism of ARIH1 in CRC. Taken together, ARIH1 is highly expressed in CRC, promoting the proliferation and metastasis of CRC cells and correlating with poor prognosis. Mechanistically, ARIH1 facilitates K63‐linked polyubiquitination of PHB1 by targeting the K186 residue, thereby enhancing its interaction with Akt. This enables PHB1 to be phosphorylated by Akt and translocated into the mitochondria, stabilizing mitochondrial dynamics and enhancing mitochondrial respiration and OXPHOS activity.

We demonstrated that ARIH1 overexpression is associated with a poorer CRC prognosis. ARIH1 plays a key role in facilitating the progression of CRC by regulating mitochondrial fusion and fission to maintain stability and promoting mitochondrial respiration and OXPHOS. Mechanistically, ARIH1 mediates the K63‐linked polyubiquitination of PHB1, thereby promoting the interaction between PHB1 and Akt, enabling PHB1 phosphorylation by Akt, and facilitating PHB1 translocation into mitochondria to exert its effects. Our findings may provide novel insights for CRC diagnosis and the identification of rational drug targets for CRC treatment (**Figure** **8**).

## 5 Experimental Section

### Clinical Samples and Tissue Microarray (TMA)

Eighty pairs of colorectal tumors and adjacent normal tissues were collected from 80 patients who underwent laparoscopic radical resection at the First Affiliated Hospital of Nanjing Medical University (Nanjing, China) between 2017 and 2020. No patients had received chemotherapy, radiotherapy, or other interventions before undergoing surgery. Written informed consent was obtained from all participants. Liquid nitrogen or −80 °C refrigerator was used to temporarily or permanently preserved the fresh tissues collected during surgery. A formalin‐fixed paraffin‐embedded (FFPE) tissue microarray containing specimens from 80 patients was constructed by Servicebio (Wuhan, China), and complete medical records of these patients were obtained for further analysis. The study was approved by the Ethics Committee of the First Affiliated Hospital of Nanjing Medical University.

### Cell Culture

The human normal colonic epithelial cell line NCM460, HEK293T, along with CRC cell lines, including HCT116, DLD‐1, SW620, SW480, LOVO, and RKO, were purchased from Servicebio Technology (Wuhan, China). Fetal bovine serum and culture media were obtained from Thermo Fisher Scientific (USA). All the cells were cultured in complete culture medium containing 10% fetal bovine serum and 1% antibiotics (penicillin and streptomycin) under 5% CO_2_ at 37 °C.

### RNA Extraction and Quantitative Real‐Time PCR (qRT‐PCR)

According to the manufacturer's protocol, TRIzol solution (Invitrogen, USA) was used to extract total RNA, the purity and concentration of which were determined via spectrophotometry. Reverse transcription was performed using an RNA reverse transcription kit (Vazyme, China). Then, qRT‐PCR was performed following the instructions for the qRT‐PCR kit (Vazyme, China).

### Plasmid Construction and Lentiviral Transfection

The lentivirus and plasmids used for ARIH1 overexpression or knockdown were designed and constructed by Genomeditech (China). The wild‐type and mutant plasmids of Flag‐tagged‐ARIH1 and Myc‐tagged‐PHB1, along with HA‐tagged‐ubiquitin were constructed by Genomeditech (China). The constructed plasmid was transiently transfected into cells using Lipofectamine 8000 reagent (Beyotime Biotechnology, China). After 48 h of plasmid transfection and lentiviral transduction, target cells were selected with puromycin (2 µg/mL). To verify the transfection efficiency, qRT‐PCR and Western blot analyses were performed.

### Cell Proliferation Assays

The 5‐ethyl‐2′‐deoxyuridine (EdU) incorporation assay kits and Cell Counting Kit‐8 (CCK‐8) were purchased from Beyotime (Shanghai, China). CCK‐8 assays, colony formation experiments, and EdU incorporation tests were conducted according to previously described methods.^[^ ^51^, ^52^ ^]^ The experiments were repeated three times.

### Cell Migration Assays

The Transwell chambers were obtained from Corning (USA). Wound healing assays and Transwell migration and invasion assays were performed as previously reported.^[^ ^52^ ^]^ Three random fields were selected and assessed via microscopic analysis.

### Co‐Immunoprecipitation (Co‐IP) Assay

Co‐IP experiments were conducted as previously reported.^[^ ^53^ ^]^ An IP/Co‐IP kit (#88 804) was purchased from Thermo Fisher Scientific. Western blotting (WB) or mass spectrometry (BGI Shenzhen, Guangdong, China) was performed to analyze the immunoprecipitated proteins.

### WB

Proteins were extracted from cells using radioimmunoprecipitation (RIPA) buffer (Beyotime, China), and protein concentrations were quantified using a bicinchoninic acid (BCA) protein assay kit (Beyotime, China). The reagents used in these experiments, such as 10% SDS‐PAGE, antibody dilution buffer, and blocking solution, were obtained from Beyotime Biotechnology, China. The WB results were subsequently obtained through a series of steps, including sample loading, electrophoresis, membrane transfer, blocking, washing, antibody incubation, and exposure imaging. The details of the antibodies used in the study are provided in Table S1 (Supporting Information). Following antibody incubation, the PVDF membrane was washed using a five‐cycle procedure, each lasting 10 min, with TBST containing 0.1% Tween‐20. The blots were subsequently visualized using a Tanon‐4600 Chemiluminescence Imaging System (Model: Tanon‐4600), which was set to the “Auto‐Exposure” mode.

### Nuclear‐Cytoplasmic Extraction and Mitochondrial Isolation

The nuclear and cytoplasmic protein fractions and the mitochondrial fraction were extracted using the Nuclear and Cytoplasmic Protein Extraction Kit and the Cell Mitochondria Isolation Kit respectively (Beyotime, China), following the manufacturer's protocol.

### Immunohistochemistry (IHC)

Samples, including both human colorectal tissues and subcutaneous tumor and liver metastasis tissues from mice, were subjected to formalin fixation and paraffin embedding for IHC analysis. IHC was performed as previously reported.^[^ ^54^ ^]^ The staining intensity and percentage of stained cells were scored as follows: 0 (negative, 0%), 1 (weak, 1–24%), 2 (moderate, 25–49%), 3 (strong, 50–74%), and 4 (75–100%). The relative expression levels of genes were assessed according to the histochemistry scores (H‐scores).

### Immunofluorescence (IF) Assay

The IF experiments were carried out according to previously described methods.^[^ ^55^ ^]^ The samples were stained with DAPI for nuclear staining (blue) and with MitoTracker Deep Red FM for mitochondrial staining (red). Confocal microscopy (Carl Zeiss) was performed to detect green foci representing PHB1.

### MitoTracker Red

A MitoTracker Red Kit (Invitrogen, USA) was used to assess mitochondrial membrane potential. The research was performed as previously described.^[^ ^55^ ^]^ Cell observations were performed via a fluorescence microscope from Leica Microsystems CMS Gmbh (Ernst‐Leitz‐Str).

### Electron Microscopy

CRC cells were fixed in 2.5% glutaraldehyde and then examined and photographed using a transmission electron microscope (Hitachi H‐500 electron microscope, FEI, USA).

### Cellular Energy Metabolism

A Seahorse XF cell energy metabolism phenotype assay kit (Agilent, USA) was used to measure the oxygen consumption rate (OCR) of CRC cells. Approximately 4 × 10^5^ cells per well were inoculated into XFe96 cell culture plates the day before the experiment. The experimental process followed the manufacturer's protocol. Oligomycin (1.5 µm), FCCP (0.5 µm), and rotenone/antimycin A (0.5µM) were added to the A, B, and C dosing chambers, respectively. A Cell Energy Metabolism Analyzer (Seahorse XFe96, Agilent, USA) and Wave software were used to calculate the key parameters that reflect mitochondrial function (basal respiration, maximal respiration, and ATP production) and analyze them.

### Animal Models

In this study, 5‐week‐old male BALB/c nude mice were used to establish models of subcutaneous tumor formation and liver metastasis. A total of 100 µL cell suspensions containing 1 × 10^6^ DLD‐1 cells transfected with shNC or shARIH1 or SW480 cells transfected with the vector or ARIH1‐oe were subcutaneously rejected into the of the left or right groin of the mice. At 25 days post‐injection, the mice were sacrificed; the tumors were measured to assess volume, weighed every 5 days, and dissected for final measurement and H&E and IHC staining. For the liver models, an equivalent dose of the treated cell suspension was injected into the distal tip of the spleen. Four weeks after injection, the mice received an intraperitoneal injection of D‐luciferin (150 mg kg^−1^) (Goldbio, USA). Metastases were visualized 10 min later with an IVIS 100 Imaging System (Xenogen, USA). The mice were euthanized, and their livers were excised. The collected tissue samples were subjected to H&E and IHC staining after imaging. All the animal experiments were approved by the Animal Ethics Committee at Nanjing Medical University (IACUC‐2308042).

### Statistical Analysis

GraphPad Prism 9.0 (La Jolla, USA) and SPSS 25.0 (Chicago, USA) were used for data analysis. Statistical analyses included t‐tests, chi‐square (χ^2^) tests, Kaplan‐Meier survival analysis, and one‐way analysis of variance (ANOVA) to evaluate differences between different samples. Given that the clinicopathological data were categorical variables, contingency tables and either the χ^2^ test or two‐tailed Fisher's exact test were employed to compare them. Survival curves for 80 patients in the tissue microarray were generated using Kaplan‐Meier survival analysis. Data from cell lines and animal experiments were analyzed using either student's t‐test or ANOVA. The student's t‐test was used to analyze the difference between two samples, whereas ANOVA was employed for comparisons involving more than two groups. Cell counting and statistical analysis were performed using ImageJ software, and each experiment was repeated more than three times. Statistical significance was defined as *p* ≤0.05.

### Ethics Approval

This study was conducted in accordance with the principles of the Declaration of Helsinki and was approved by the Ethics Committee of the First Affiliated Hospital of Nanjing Medical University (2024‐SR‐361). All animal experiments were approved by the Animal Ethics Committee of Nanjing Medical University (IACUC‐2308042)

### Consent for Publication

All authors have provided their consent to publish the manuscript.

## Conflict of Interest

The authors declare no conflict of interest.

## Author Contributions

Ying Tong (Y.T.), Zhenling Wang (Z.W.), Yong Wang (Y.W.), and Yang Chen (Y.C.) contributed equally to this work. Zan Fu (Z.F.) was the primary investigator in this study, conceptualizing the study, obtaining financial backing, and supervising the entire study. Y.T., Y.W., and Z.W. devised the project. Y.T., Z.W., and Y.C. carried out the experiments and drafted the initial manuscript. Y.T., Y.C., and Yunfei Lu (Y.L.) contributed mouse experiments. Y.T., Y.W., and Hongqiang Zhang (H.Z.) performed statistical analyses. Z.W., Y.W., and Y.C. revised the manuscript. All authors were responsible for collecting and organizing CRC samples. Y.C., Y.L., and Lei Xu(L.X.) supplied support with experimental and clinical techniques. After a careful examination, all authors approved the manuscript's final draft.

## Supporting information



Supporting Information

Supplemental Table3

## Data Availability

The data that support the findings of this study are available on request from the corresponding author. The data are not publicly available due to privacy or ethical restrictions.
