# Supplementary material for: The E3 Ubiquitin Ligase ARIH1 Facilitates Colorectal Cancer Progression by Promoting Oxidative Phosphorylation via the Mitochondrial Translocation of K63‐Linked Ubiquitinated PHB1
Source: Adv Sci (Weinh). 2025 Apr 26;12(25):2501017. doi: 10.1002/advs.202501017 (PMC12224947; doi:10.1002/advs.202501017)
Supplement: Supplementary file 1 — Supporting Information [file ADVS-12-2501017-s001.docx]

**Table S1. Antibodies used in this study.**

| **Antigens** | **Manufacturer** | **Application** |
| --- | --- | --- |
| ARIH1 | Proteintech No.14949-1-AP | WB/IHC/IP |
| PHB1 | Proteintech No.10787-1-AP | WB/IP/IF |
| α-tubulin | Proteintech No.11224-1-AP | WB |
| HDAC1 | Proteintech No.10197-1-AP | WB |
| VDAC1 | Proteintech No.10866-1-AP | WB |
| Ubiquitin | Santa Cruz Biotechnology sc-8017 | IP/ WB |
| FLAG | Proteintech No.20543-1-AP | IP/ WB |
| HA | Proteintech No.51064-2-AP | IP/ WB |
| His | Proteintech No.66005-1-Ig | IP/ WB |
| Myc | Proteintech No.60003-2-Ig | IP/ WB |
| GST | Proteintech No.66001-2-Ig | pull down |
| Goat Anti-Mouse IgG | Proteintech No.SA00001-1 | WB |
| Goat Anti-Rat IgG | Proteintech No.SA00001-15 | WB |
| Anti-Rat alexa fluor 488 | Beyotime P0176 | IF |
| Ki67 | Servicebio GB111499 | IHC |
| Akt | Proteintech No.10176-2-AP | IP/ WB |
| OPA1 | Proteintech No.27733-1-AP | WB |
| MFN1 | Proteintech No.13798-1-AP | WB |
| DRP1 | Proteintech No.12957-1-AP | WB |
| FIS1 | Proteintech No.10956-1-AP | WB |
| IgG | Abcam ab172730 | IP |
| Phospho-PHB1 | Abcam ab117253 | IP/ WB |
| Phospho-Akt | Abcam ab38449 | WB |
| Phospho-GSK3β | Proteintech No.14850-1-AP | WB |

**Table S2. Primers, shRNAs used in the study.**

| **qRT-PCR Primers (5’-3’)** | | |
| --- | --- | --- |
| ARIH1 | Forward primer | GTCTCGAACACGCCAGATGAATA |
|  | Reverse primer | TGACCCATGCCTTCTTCCATTAT |
| GAPDH | Forward primer | GGACCTGACCTGCCGTCTAG |
|  | Reverse primer | GTAGCCCAGGATGCCCTTGA |
| PHB1 | Forward primer | AAACAGGTGGCTCAGCAGGAA |
|  | Reverse primer | CAGTGAGTTGGCAATCAGCTCAG |
| **Short hairpin RNAs sequences** | | |
| shNC | sense | GATCTGTTCTCCGAACGTGTCACGTTTCAAGAGAACGTGACACGTTCGGAGAATTTTTTC |
|  | antisense | AATTGAAAAAATTCTCCGAACGTGTCACGTTCTCTTGAAACGTGACACGTTCGGAGAACA |
| shARIH1-1 | sense | GATCCGCTACCTTGAACGAGATATTTCTCGAGAAATATCTCGTTCAAGGTAGCTTTTTT |
|  | antisense | AATTAAAAAAGCTACCTTGAACGAGATATTTCTCGAGAAATATCTCGTTCAAGGTAGCG |
| shARIH1-2 | sense | GATCCGCCATGTTGTTAAAGTCCAATACTCGAGTATTGGACTTTAACAACATGGTTTTTT |
|  | antisense | AATTAAAAAACCATGTTGTTAAAGTCCAATACTCGAGTATTGGACTTTAACAACATGGCG |
| shARIH1-3 | sense | GATCCGAACTACCCTAACTCGTATTTCTCGAGAAATACGAGTTAGGGTAGTTCTTTTTT |
|  | antisense | AATTAAAAAAGAACTACCCTAACTCGTATTTCTCGAGAAATACGAGTTAGGGTAGTTCG |

**Table S3. The H-score of ARIH1 and the clinicopathological information of 80patients**

Please refer to Table S3 in the Excel file.

**
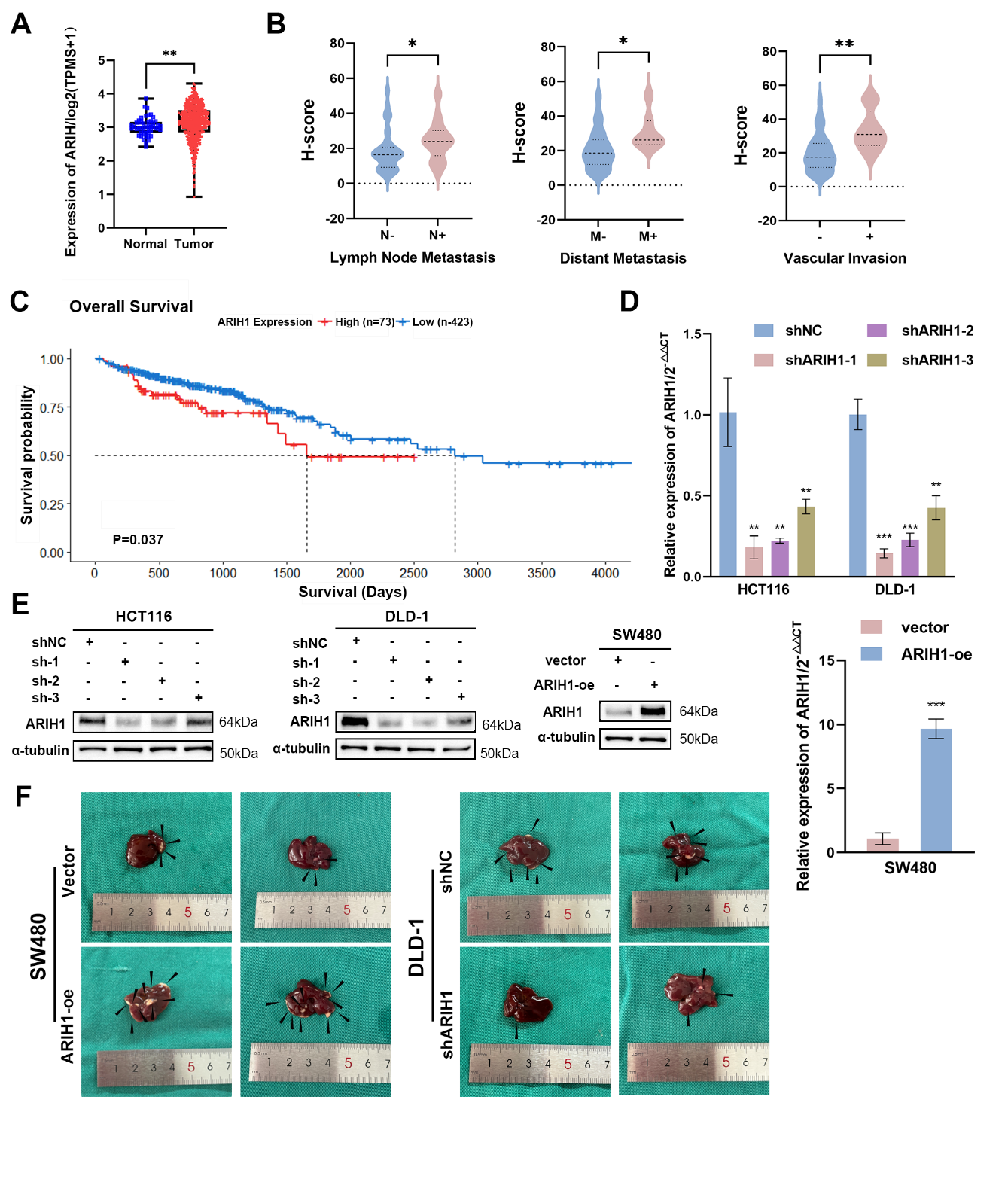
**

**Supplementary Figure 1. A** Differences in mRNA expression of ARIH1 between normal (n=48) and various tumor cells (n=541) in TCGA-COAD and TCGA-READ cohort. **B** Differences in H-score among different clinicopathological features. **C** The Kaplan-Meier survival analysis of patients with high (n=73) and low (n=423) ARIH1 expression based on TCGA data. **D,E** qRT-PCR and western blotting were used to measure the knockdown and overexpression efficiency of ARIH1 in selected CRC cell lines. **F** Representative liver images of different groups obtained from nude mice. The lesion indicated by the arrow represents a metastatic deposit in the liver. All data was shown as mean±SD of three independent experiments and was analyzed using either student's t-test or ANOVA, *P<0.05, **P<0.01, ***P<0.001.


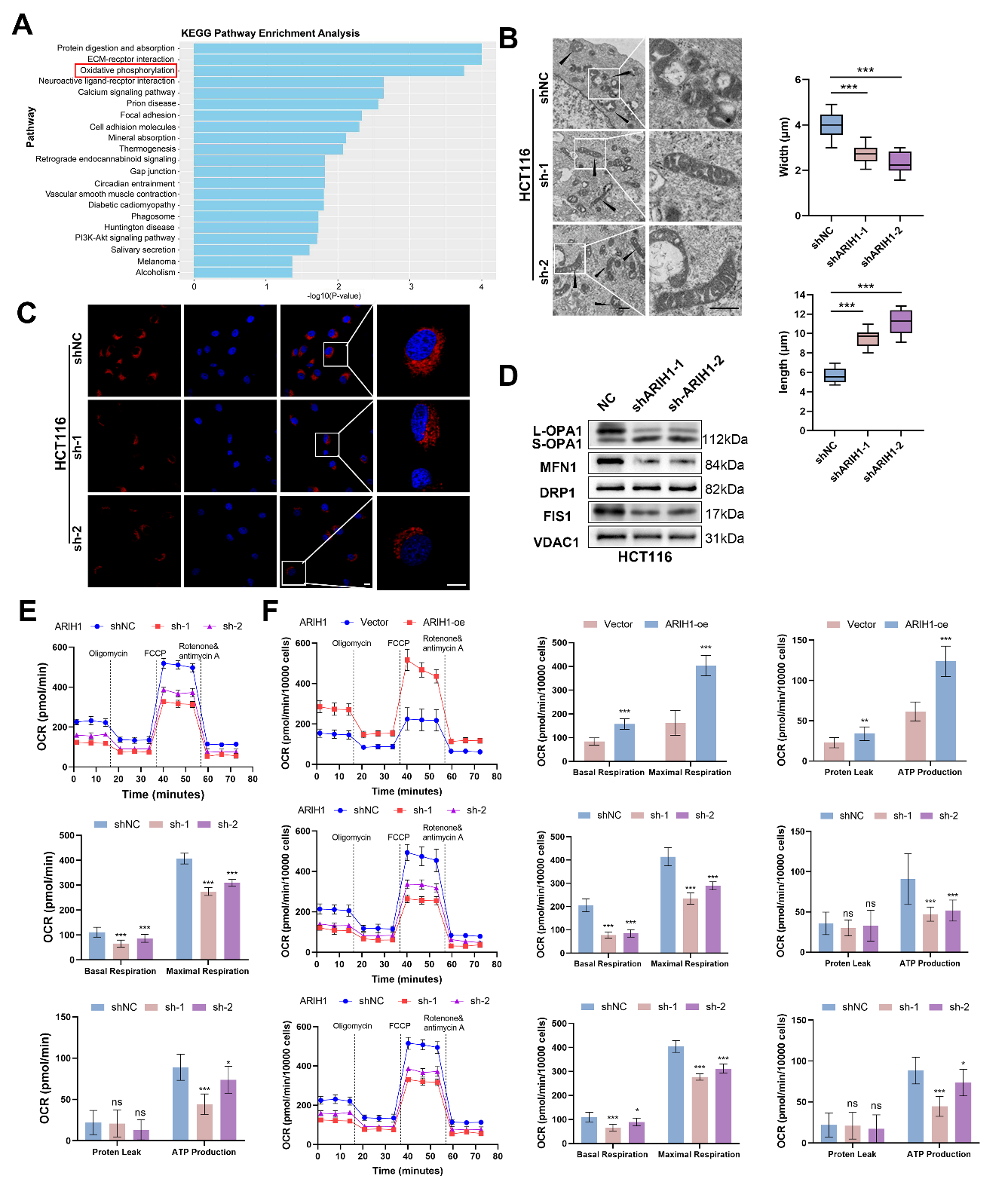


**Supplementary Figure 2. A** Representative KEGG enrichment analysis between groups expressing high and low levels of ARIH1 based on TCGA data revealed that ARIH1 was mainly involved in oxidative phosphorylation pathways. **B** The morphology of mitochondria in HCT116 cells with ARIH1 knockdown was assessed using electron microscopy, and the width and length of mitochondria were quantified and statistically analyzed. Black scale bar: 0.5μm. **C** Representative images of MitoTracker Red assay in HCT116 cells with ARIH1 knockdown to assess mitochondrial membrane potential. White scale bar: 10μm. **D** Western blot analysis pf fusion (OPA1, MFN1) and fission (DRP1, FIS1) in HCT116 cells. **E** The oxygen consumption rate (OCR) was measured with the Seahorse XF96 Analyzer in CRC cells applying mitochondrial stress test conditions. Basal respiration, maximal respiration, proton leak, and ATP production were measured and calculated. **F** Normalized OCR, basal respiration, maximal respiration, proton leak, and ATP production were calculated following the quantification of each well in the experiment. All data was shown as mean±SD of three independent experiments and was analyzed using either student's t-test or ANOVA, *P<0.05, **P<0.01, ***P<0.001.


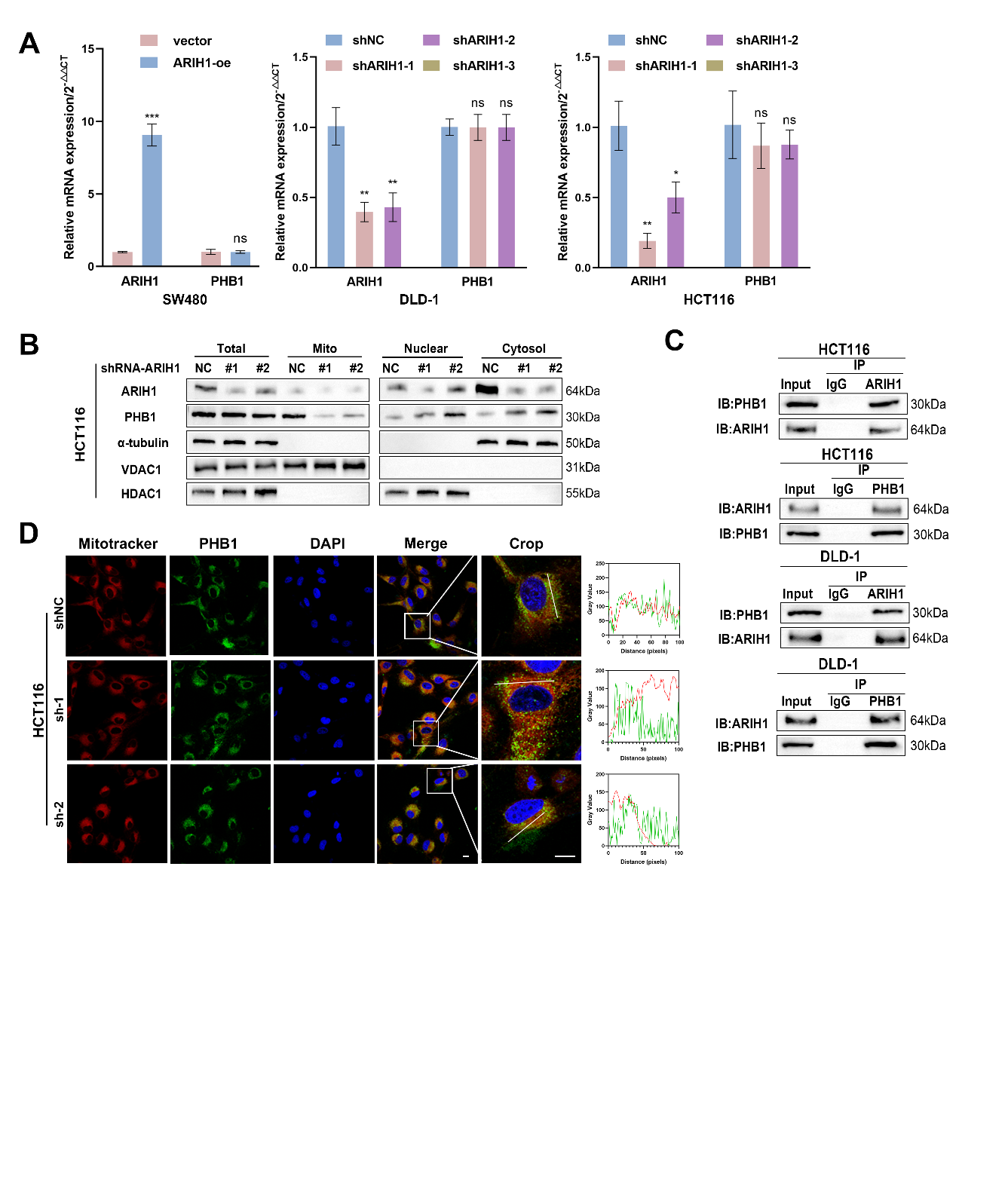


**Supplementary Figure 3. A** The mRNA levels of PHB1 in SW480 cells overexpressing ARIH1 and DLD-1 and HCT116 cells with ARIH1 knockdown by q-PCR. **B** Protein levels of PHB1 in whole cells, nucleus, cytoplasm (excluding mitochondria), and mitochondria extracts were assessed by Western blotting in HCT116 cells tranfected with shRNAs. α-tubulin, VDAC1, and HDAC1 served as a loading control. **C** Cytoplasmic extracts (excluding mitochondria) from DLD-1 and HCT116 cells were analyzed by Co-IP followed by Western blot analysis to investigate the endogenous interaction between ARIH1 and PHB1. **D** Representative images of PHB1 and MitoTracker Deep Red FM immunofluorescence staining in HCT116 cells obtained to assess the co-localization of PHB1 with the mitochondrial marker. Intensity profiles (right panel) were derived from the cropped images, indicated by a small white line. Red fluorescence corresponds to the mitochondrial marker, while green fluorescence denotes PHB1. Scale bar: 10μm. All data was shown as mean±SD of three independent experiments and was analyzed using either student's t-test or ANOVA, *P<0.05, **P<0.01, ***P<0.001.


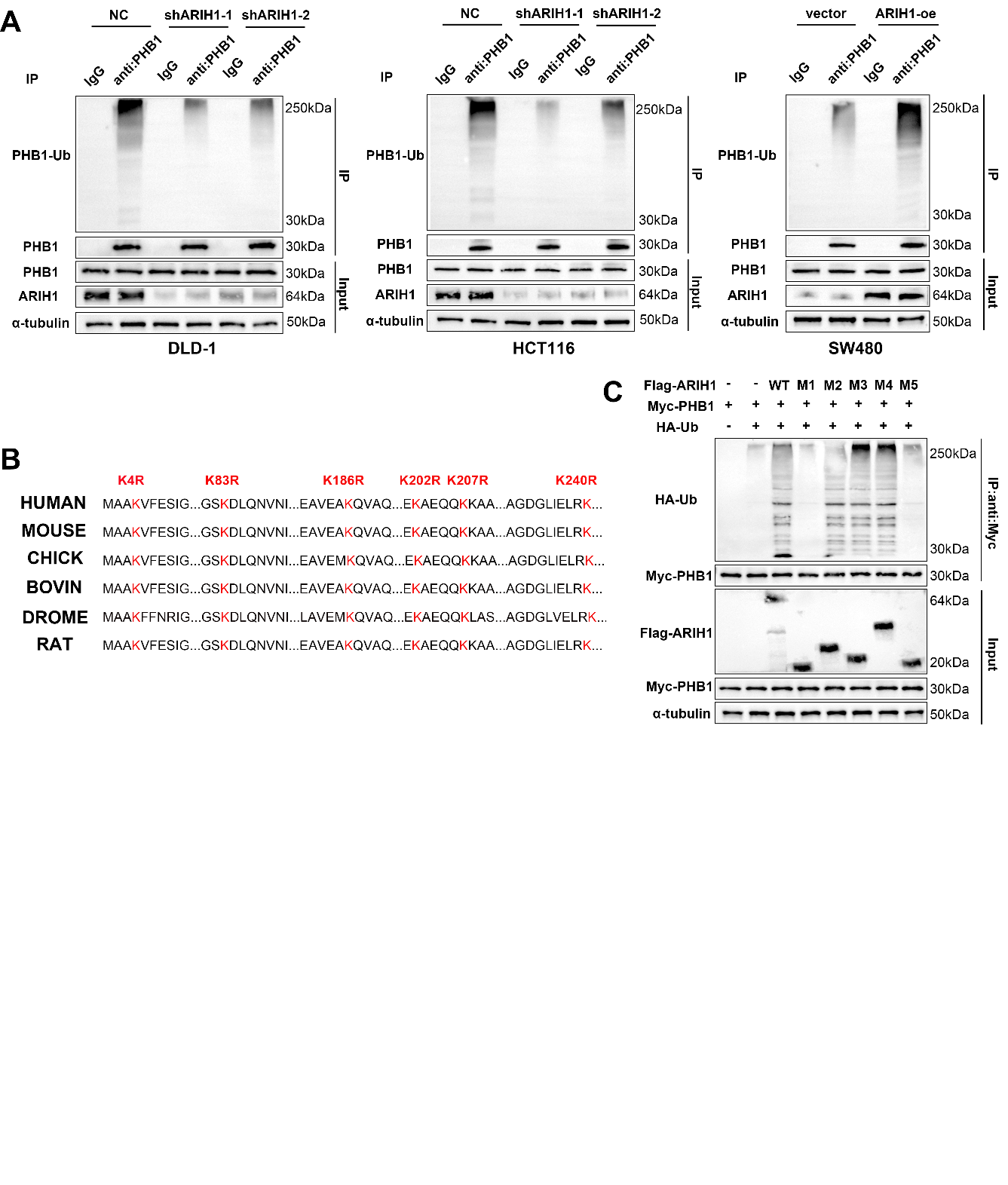


**Supplementary Figure 4. A** SW480 cells transfected with either an empty vector or an ARIH1 overexpression lentivirus, as well as DLD-1 and HCT116 cells transfected with shNC or shRNA lentiviral vectors, were analyzed for PHB1 ubiquitination. **B** Amino acids sequences alignment of the K4, K83, K186, K202, K207, and K240 in PHB1 from different spices. **C** Plasmids encoding Flag-ARIH1, HA-Ub, together with plasmids encoding either wild-type Myc-PHB1 or its mutants (M1, M2) were transfected into 293T cells. After 24 hours, cell lysates were harvested and the ubiquitination levels were analyzed by WB using the indicated antibodies. The results are representative of three independent experiments.


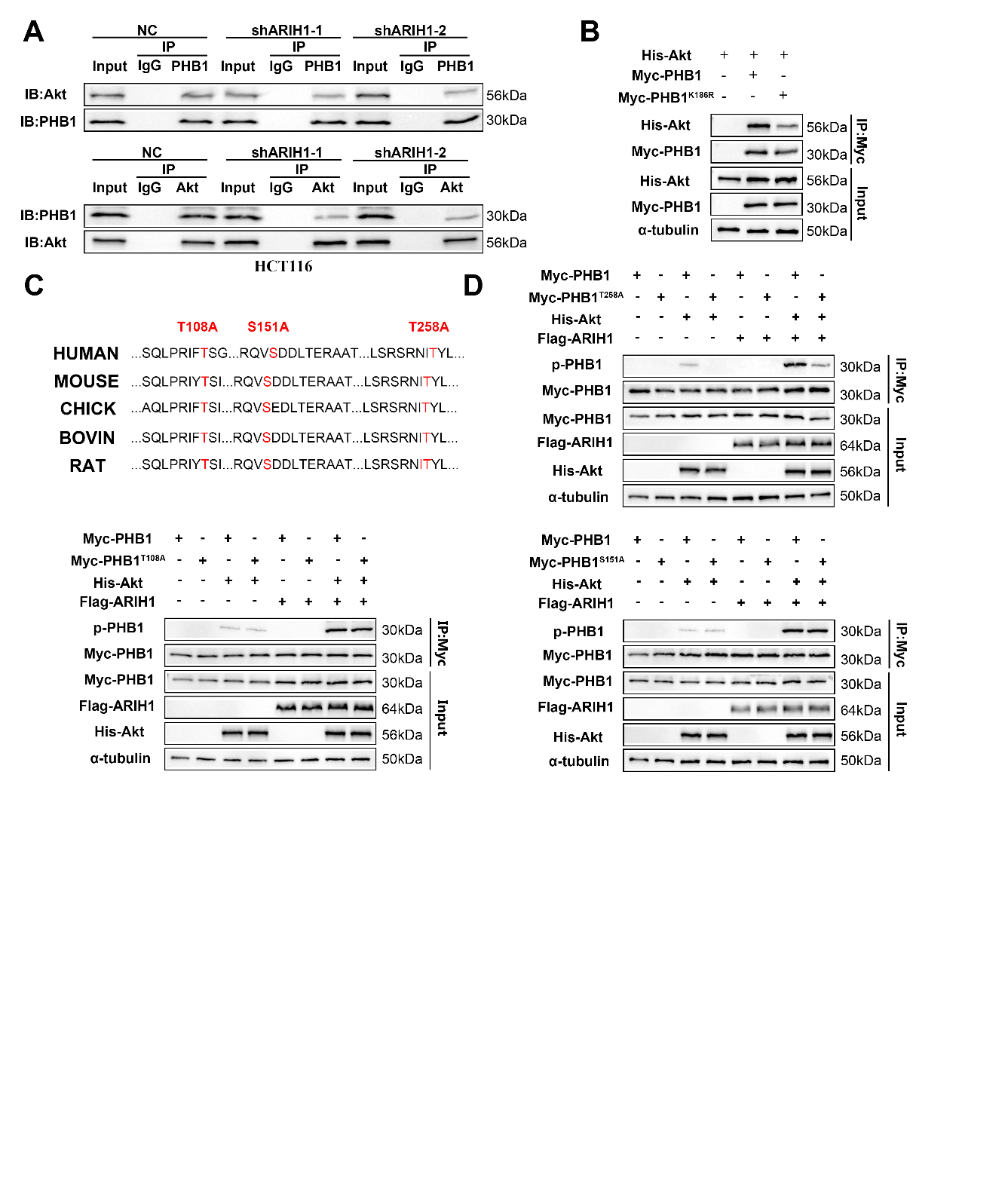


**Supplementary Figure 5. A** Lysates from HCT116 cells with ARIH1 knockdown were analyzed by Co-IP followed by WB to investigate the endogenous interaction between PHB1 and Akt. **B** Cell lysates from HEK293T cells transfected with His-Akt and either wild-type Myc-PHB1 or its mutant (Myc-PHB1^K186R^) plasmid for 24 hours were analyzed by Co-IP and WB to investigate exogenous protein interactions between PHB1 and Akt. **C** Amino acids sequences alignment of the T108, S151, and T258 in PHB1 from different spices. **D** HEK293T transfected with Myc-PHB1^T108A,^ Myc-PHB1^S151A^, Myc-PHB1^T258A^ were then lysed and Myc-PHB1^T108A,^ Myc-PHB1^S151A^, Myc-PHB1^T258A^ were immunoprecipitated and subjected to WB to detect the phosphorylation level of PHB1. The results are representative of three independent experiments.
